# New Insights Into the Relationships Within Subtribe Scorzonerinae (Cichorieae, Asteraceae) Using Hybrid Capture Phylogenomics (Hyb-Seq)

**DOI:** 10.3389/fpls.2022.851716

**Published:** 2022-07-01

**Authors:** Elham Hatami, Katy E. Jones, Norbert Kilian

**Affiliations:** ^1^Department of Biology, Faculty of Science, Shahid Bahonar University of Kerman, Kerman, Iran; ^2^Botanic Garden and Botanical Museum Berlin, Freie Universität Berlin, Berlin, Germany

**Keywords:** phylogenetics, next-generation sequencing, myBaits COS compositae 1Kv1, plastome, multispecies coalescent model, taxonomy, *Scorzonera*

## Abstract

Subtribe Scorzonerinae (Cichorieae, Asteraceae) contains 12 main lineages and approximately 300 species. Relationships within the subtribe, either at inter- or intrageneric levels, were largely unresolved in phylogenetic studies to date, due to the lack of phylogenetic signal provided by traditional Sanger sequencing markers. In this study, we employed a phylogenomics approach (Hyb-Seq) that targets 1,061 nuclear-conserved ortholog loci designed for Asteraceae and obtained chloroplast coding regions as a by-product of off-target reads. Our objectives were to evaluate the potential of the Hyb-Seq approach in resolving the phylogenetic relationships across the subtribe at deep and shallow nodes, investigate the relationships of major lineages at inter- and intrageneric levels, and examine the impact of the different datasets and approaches on the robustness of phylogenetic inferences. We analyzed three nuclear datasets: exon only, excluding all potentially paralogous loci; exon only, including loci that were only potentially paralogous in 1–3 samples; exon plus intron regions (supercontigs); and the plastome CDS region. Phylogenetic relationships were reconstructed using both multispecies coalescent and concatenation (Maximum Likelihood and Bayesian analyses) approaches. Overall, our phylogenetic reconstructions recovered the same monophyletic major lineages found in previous studies and were successful in fully resolving the backbone phylogeny of the subtribe, while the internal resolution of the lineages was comparatively poor. The backbone topologies were largely congruent among all inferences, but some incongruent relationships were recovered between nuclear and plastome datasets, which are discussed and assumed to represent cases of cytonuclear discordance. Considering the newly resolved phylogenies, a new infrageneric classification of *Scorzonera* in its revised circumscription is proposed.

## Introduction

**FIGURE 1:**
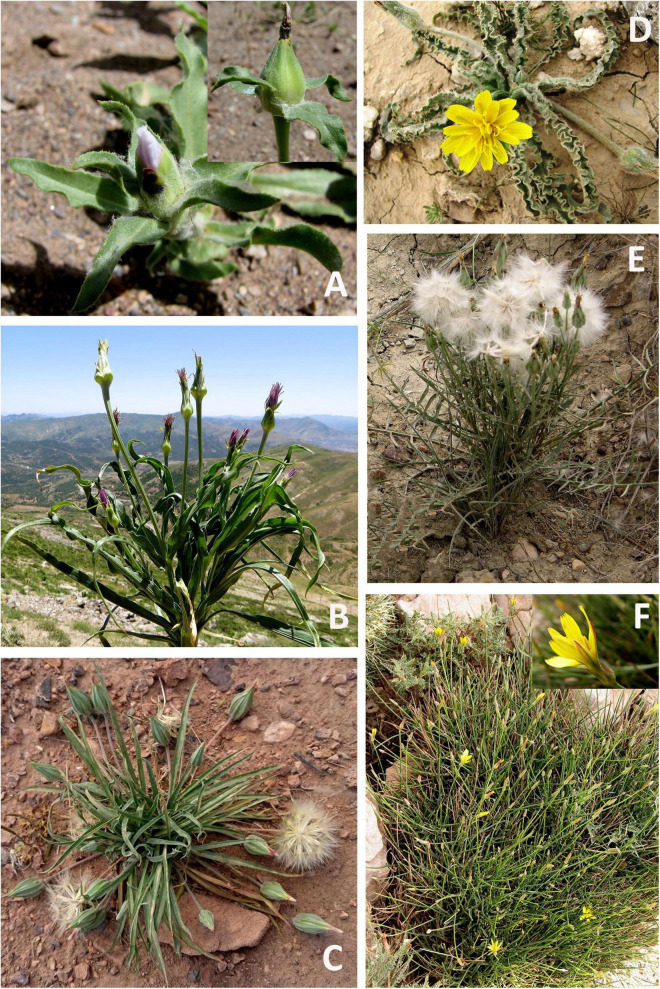
Diversity in Scorzonerinae shown by photographs of representative species. For each image, locality, and date of the photograph are given. **(A)** *Epilasia acrolasia*, Iran, Kerman, Rafsanjan, Khenaman village, 19 April 2007; **(B)** *Pseudopodospermum phaeopappum*, Iran, Kordestan, Baneh, May 2019; **(C)** *Pterachaenia stewartii*, Iran, Kerman, near Dehbala village, 15 May 2019; **(D)** *Gelasia lanata*, Iran, Khuzistan, 11 km from Bagh-Malek to Meidavud, 9 March 2007; **(E)** *Scorzonera persepolitana*, Iran, Esfahan, near Delijan, on clay hill, 20 May 2019; and **(F)** *Scorzonera rupicola*, Iran, Bakhtiari, between Esfahan and Shahrekord, 2 July 2010. All photographs by M. Mirtadzadini.

The Scorzonerinae Dumort., recognized as a subtribe of the tribe Cichorieae in the hyperdiverse angiosperm family Asteraceae or Compositae, include some 300 species that are chiefly native to Europe, North Africa, and extratropical Asia (Kilian et al., 2009a,b; Figure 1). The subtribe has been confirmed as monophyletic in phylogenetic analyses based both on morphological and molecular data. It is characterized by a unique plumose pappus type (by reversal rarely reduced or missing) and equally unique pollen with only bilacunar colpori, of which an unparalleled diversity of distinctive pollen types has evolved (Blackmore, 1986; Bremer, 1994; Mavrodiev et al., 2004; Tremetsberger et al., 2012; Zaika et al., 2020). Several morphologically well-delimited entities have widely been accepted as genera in the Scorzonerinae, including *Epilasia* (Bunge) Benth., *Geropogon* L., *Koelpinia* Pall., *Pterachaenia* (Benth.) Stewart, *Scorzonera* L., *Tourneuxia* Coss., and *Tragopogon* L. (Bremer, 1994). The circumscription of the name-giving genus *Scorzonera* has, however, been a subject of taxonomic debate almost ever since its establishment, and no less than six segregates had been proposed by 1990, but only two, *Podospermum* DC. and *Pseudopodospermum* Kuth., gained some recognition, although mostly at subgeneric rank only (Zaika et al., 2020). Molecular phylogenetic studies then indicated that *Scorzonera* in its traditional wide sense (s.l.) is a polyphyletic assemblage and that its various clades are completely intermingled in phylogenetic trees with the traditionally accepted genera (Whitton et al., 1995; Mavrodiev et al., 2004, 2012; Winfield et al., 2006; Kilian et al., 2009a; Hatami et al., 2020; Zaika et al., 2020). The most comprehensive study to date, by Zaika et al. (2020), demonstrated that most of the proposed segregates represent diverging lineages of *Scorzonera* s.l., although often with a surprisingly different circumscription. Zaika et al. (2020) even identified two additional divergent lineages of the *Scorzonera* s.l. and, therefore, established two new genera. Although individual lineages of the Scorzonerinae (Figure 1) were well-supported in previous phylogenetic analyses, these studies fell short in resolving the relationships in the subtribe, because the deeper nodes of the phylogenetic tree of the Scorzonerinae remained unresolved due to the limited phylogenetic signal of the applied Sanger sequencing markers. This holds similarly for interspecific relationships within the lineages also because many shallow nodes remained unresolved. In the last decade, next-generation sequencing technologies have emerged as an important methodological advance for resolving the phylogeny of taxonomically complex groups at different evolutionary levels and have given researchers the ability to produce massive amounts of genomic data across many taxa at affordable costs (Harrison and Kidner, 2011; Buggs et al., 2012; Godden et al., 2012; Stoughton et al., 2018). Among the genome-scale methods developed to date, hybrid capture [also termed target(ed) capture or target enrichment] of single or low copy sequences combined with high-throughput sequencing, also known as Hyb-Seq, is the most efficient and cost-effective approach for obtaining large datasets of single-copy nuclear genes for plant systematics, allowing studies at different evolutionary scales and, of great importance, efficiently recovering sequences also from degraded DNA extracted from old museum specimens (Cronn et al., 2012; Lemmon et al., 2012; Mandel et al., 2014; Weitemier et al., 2014; Dodsworth et al., 2019; Forrest et al., 2019). Hyb-Seq technique uses short RNA or DNA probes designed across the taxonomic group of interest from known sequence data and used as “baits” to capture the target loci from fragmented genomic DNA libraries by hybridization reactions (Mamanova et al., 2010; Lemmon et al., 2012; McCormack et al., 2013; Buddenhagen et al., 2016). Regarding the targeted loci, it is critical to discriminate, for any individual samples, orthologs from paralogs, because paralogous sequences can bias the phylogenetic inference. A common approach to account for paralogy is removing the loci that show evidence of potential paralogy (Mandel et al., 2014; Weitemier et al., 2014; Chamala et al., 2015; Schmickl et al., 2016; Folk et al., 2017; Emms and Kelly, 2019; Glover et al., 2019; Jones et al., 2019; Andermann et al., 2020; Fernández et al., 2020). Further investigation of duplicate loci flagged as paralogs can be rewarding because, in some loci, the duplicates may not be paralogs but represent allelic variation (Johnson et al., 2016). More importantly, in allopolyploids orthology, inference among duplicated loci (then being homoeologs, Glover et al., 2016) can be used to establish the origin of such lineages (Yang and Smith, 2014; Morales-Briones et al., 2021). Depending on the design of the targets, the Hyb-Seq approach has the additional advantage of recovering not only targeted sets of loci that are highly conserved exons, but also parts of the more variable and flanking non-coding sequences (introns and intergenic spacers), hence, producing “supercontigs,” which can be aligned and analyzed together to amplify the phylogenetic signal (Weitemier et al., 2014; Johnson et al., 2016; Jones et al., 2019; Bagley et al., 2020; Gardner et al., 2021). Moreover, this approach also allows the recovery of plastome data from off-target sequenced reads, enabling an independent estimate of phylogeny and inference from a principally and maternally inherited genome (Weitemier et al., 2014; Mandel et al., 2015; Dillenberger et al., 2018; Herrando-Moraira et al., 2019). For Compositae, Mandel et al. (2014) developed a probe set of exons of 1,061 orthologous loci, available as myBaits COS Compositae 1Kv1. This “conserved ortholog set” (COS) for Compositae has been further explored by Mandel et al. (2017); Jones et al. (2019), and Siniscalchi et al. (2021), and successfully applied to provide a well-resolved family backbone (Mandel et al., 2019), as well as resolving phylogenetic relationships in several difficult groups at different evolutionary levels (Herrando-Moraira et al., 2018, 2019, 2020; Siniscalchi et al., 2019; Lichter-Marck et al., 2020; Thapa et al., 2020; Watson et al., 2020; Xu and Chen, 2021).

Here, we apply the Hyb-Seq method and the Compositae 1,061 nuclear loci set on representative samples across the major lineages of the Scorzonerinae. The main goals of the present study were: (1) To evaluate the potential of the Hyb-Seq approach for resolving the phylogenetic relationships at inter- and intrageneric levels across the subtribe; (2) To test the hypotheses on phylogenetic lineages within the Scorzonerinae inferred through the molecular phylogenetic study based on nrITS and two plastid DNA markers in addition to comparative morphology and fruit-anatomy by Zaika et al. (2020) and in this way reassess their taxonomic conclusions; (3) To examine the impact of different datasets (targeted exons in locus sets of different sizes, exons with flanking intron regions, and off-target plastome sequences) and analysis methods [multispecies coalescent, maximum likelihood (ML), and Bayesian analyses of concatenated loci] on phylogenetic reconstructions at deep and shallow nodes; (4) To explore the discordance between nuclear and plastid DNA trees; and (5) To revise the taxonomy of the Scorzonerinae at generic and infrageneric levels in light of the new results.

## Materials and Methods

### Taxon Sampling, Biological Material, and Sequence Data

Our taxon sampling scheme covered all major lineages of the subtribe Scorzonerinae as recognized in recent molecular phylogenetic studies (Hatami et al., 2020; Zaika et al., 2020), and had the aim to include a wide range of species of *Scorzonera* in its former widest sense (Zaika et al., 2020). Consequently, representatives of all described genera based on the taxonomic treatment of Zaika et al. (2020) were included: *Epilasia*, *Gelasia* Cass., *Geropogon*, *Koelpina*, *Lipschitzia* Zaika et al., *Pseudopodospermum* (Lipsch. & Krasch.) Kuth., *Pterachaenia*, *Ramaliella* Zaik et al., *Scorzonera*, *Takhtajaniantha* Nazarova, and *Tragopogon*. However, for *Tourneuxia variifolia* Coss., the only member of *Tourneuxia*, and *Lipschitzia divaricata* (Turcz.) Zaika et al., the only member of that genus, wet lab treatment did not yield sufficient reads for analysis. A total number of 152 samples from representatives of subtribe Scorzonerinae were included (Appendix). The outgroup to the Scorzonerinae included eight representatives of selected subtribes of different phylogenetic distances according to Kilian et al. (2009a) and Tremetsberger et al. (2012): Chondrillinae [*Willemetia stipitata* (Jacq.) Dalla Torre, *Chondrilla ramosissima* Sm.], Cichoriinae (*Cichorium intybus* L.), Hypochaeridinae [*Hypochaeris achyrophorus* L., *Leontodon tingitanus* Ball., *Urospermum dalechampii* (L.) F. W. Schmidt], Lactucinae (*Lactuca sativa* L.), and Scolyminae (*Scolymus hispanicus* L., *Catananche arenaria* Coss. & Durieu; Appendix).

The DNA was isolated in most cases directly from herbarium specimens of the selected taxa, or silica-dried leaf material vouchered by a corresponding herbarium specimen (12 samples). Herbarium specimens with the permission for DNA extraction for this study were kindly provided by the following herbaria: Botanic Garden and Botanical Museum Berlin (B), Ferdowsi University of Mashhad (FUMH), Ernst-Moritz-Arndt-Universität Greifswald (GFW), Komarov Botanical Institute of Russian Academy of Science St. Petersburg (LE), Staatliche Naturwissenschaftliche Sammlungen Bayerns, München (M), Shahid Bahonar University of Kerman (MIR), Ludwig-Maximilians-Universität München (MSB), Moscow State University (MW), and Naturhistorisches Museum Wien (W). Appendix also includes the specimen data, links to digitized specimens, as well as the accession numbers of the INSDC (International Nucleotide Sequence Database Collaboration) for the deposited sequence data.

### DNA Extraction, Library Preparation, Hybrid Capture, and Sequencing

Wet laboratory work was undertaken in the Botanic Garden and Botanical Museum Berlin molecular lab and the Berlin Center for Genomics in Biodiversity Research (BeGenDiv) consortium genomics lab. Genomic DNA was extracted from dried leaf tissues, using the NucleoSpin Plant II kit (Macherey-Nagel GmbH, Düren, Germany) and DNeasy Plant Mini Kit (Qiagen, Hilden, Germany) following the manufacturer’s protocol. The total genomic DNA quantity was measured with a Qubit 2.0 Fluorometer (Life Technologies, Grand Island, New York, United States). The quality of genomic DNA extractions was assessed for level of fragmentation and fragment size using a 0.9% (w/v) agarose gel. A total of 1 μg of genomic DNA in 60 μL was sheared to a target average fragment size of ∼500 bp by sonicating for 55 s using a Covaris S220 (Covaris, Brighton, United Kingdom). Sonication was not carried out for well-fragmented (<600 bp) genomic DNA samples extracted from herbarium specimens.

The DNA libraries were prepared using the NEBNext Ultra II DNA Library Prep Kit for Illumina (New England Biolabs, Ipswich, MA, United States), following the standard protocol provided by the manufacturer. We followed the library preparation wet laboratory method described for the Berlin lab, provided by Jones et al. (2019: Appendix S2). However, in some cases, we used 15, 19, or 20 cycles for PCR amplification. Hybrid capture was performed using MyBaits (Arbor Biosciences, Ann Arbor, MI, United States) and the myBaits COS Compositae 1Kv1 (Mandel et al., 2014), according to the wet laboratory methods described in detail by Jones et al. (2019: Appendix S2), but either 19 or 22 PCR cycles on the last amplification step were done. In preparation for the hybrid capture reactions, usually, around 10 samples were pooled. For one sequencing run (including 76 out of the total of 163 samples), the post-capture reaction was spiked with a pre-capture reaction (ratio 3:1) before sequencing, to yield a higher number of off-target sequences. Further quality checking and sequencing were carried out either at Macrogen Inc. (South Korea) on a Hiseq X platform (300 cycles) in paired-end, high-output mode or the BeGenDiv (Berlin, Germany) on an Illumina NextSeq platform (300 cycles) in paired-end, mid-output mode.

### Raw Data Cleaning and Reference-Guided Assembly and Paralog Assessment of Conserved Ortholog Set Loci

**FIGURE 2:**
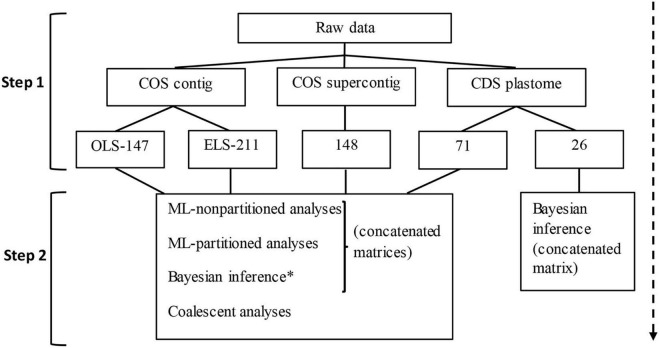
Flowchart summarizing the data assemblage (step 1) and phylogenetic analyses (step 2) in this study. Numbers in the third row correspond to the number of loci in concatenated matrices and the number of gene trees used for coalescent analyses. *only conducted for the OLS-147 dataset; COS, [nuclear] conserved ortholog set; CDS, [protein] coding sequences; OLS, original loci set; ELS, enlarged loci set; ML, maximum likelihood.

Table 1 and Figure 2 summarize the datasets assembled and their corresponding analyses. All data processing and analyses were done on the high-performance computing system of the Freie Universität Berlin (Bennett et al., 2020). Forward and reverse raw reads of each sample were subject to adapter trimming, quality filtering, and duplicate removal, using the initial data cleaning step of the HybPhyloMaker (Fér and Schmickl, 2018) pipeline, which makes use of Trimmomatic v.0.32 (Bolger et al., 2014) and FastUniq v.1.1 (Xu et al., 2012). HybPiper (Johnson et al., 2016) was used to individually map the cleaned and deduped sequences of each sample to the reference sequences of *Carthamus tinctorius*, *Helianthus annuus*, and *L. sativa* for the 1,061 loci of the COS by Mandel et al. (2014) and assemble them into contigs. To achieve this, HybPiper first searches the reads against and sorts them according to the target sequences (the individual COS loci) using BWA (Li and Durbin, 2009). The appropriate target then guides the read assembly into contigs, using SPAdes (Bankevich et al., 2012). After assembly, the SPAdes contigs are aligned to the targets, scaffolded, and translated. Finally, HybPiper extracts the sequences recovered for the same target of all samples of a giving sampling and generates an unaligned multi-FASTA file for each target. HybPiper flags loci with a paralog warning when coding sequences of more than 85% of the reference length are detected in multiple contigs. Among the competing contigs, the one is selected that has a coverage depth exceeding the other by 10×, or else the one with the greatest percent identity to the reference. Paralog flagging is particularly useful when frequent reticulation events can be suspected, but has the drawback that even simple allelic variation may trigger such warnings. Facing a high percentage of target loci with paralog warnings, we, therefore, assessed the loci with paralog warnings using the script provided by HybPiper. In this way, the inferred paralogous sequences of all loci of the sampling were collected along with all locus sequences without paralogs. The matrices for the individual loci were then aligned using MAFFT v.7.266 (Katoh and Standley, 2013) and gene trees were calculated using FastTree (Price et al., 2010). By visual inspection of these gene trees, loci possessing homologous sequences that were resolved in a sister-group relationship for typically <3 species were considered to indicate allelic variation, selected, and added back to the original loci set (OLS) without paralog warnings. To examine the effect of the additional loci gained through this step on the results of the phylogenetic analysis, the enlarged loci set (ELS) was analyzed and used separately from the OLS. We also used the HybPiper pipeline to assemble the COS exons with flanking introns (the “splash zone” of Weitemier et al., 2014). All loci with paralog warnings were removed from the matrix and the resulting dataset is hereon referred to as COS supercontigs (Table 1 and Figure 2).

**TABLE 1:** Statistics for the nuclear (OLS, ELS, and supercontig) datasets after removing loci with <50% of all samples and plastome CDS dataset after cleaning process and a summary of analyses conducted on each dataset.

Dataset	COS contigs (OLS)	COS contigs (ELS)	Supercontigs	Plastome CDS71	Plastome CDS26
Number of samples	160	160	149	124	
Number of recovered loci	147	211	148	71	26
Length of the concatenated matrix	44,634	64,358	132,646	63,917	48,478
Number/Percent of variable sites	25,431/54%	36,156/54%	89,102/67%	25,141/36%	20,226/40%
Number/Percent of parsimony informative sites	16,315/35%	23,379/35%	65,023/48%	6,321/8%	5,300/11%
Average percentage of missing data per locus (min–max)	8% (0–27.5%)	8.6% (0–27.4%)	7.5% (3.9–11.1%)	25.9% (6.6–47.4%)	25% (10–38.8%)
Average number of taxa recovered per locus (min–max)	146 (79–160)	147 (83–160)	96 (66–133)	124 (124–124)	124 (124–124)
Average sequence length per locus (min–max)	304 (69–772)	305 (69–773)	896 (426–1,978)	900 (78–6,840)	1,795 (390–6,840)
Type of analysis conducted on concatenated matrix	1-ML unpartitioned analysis 2-ML partitioned analysis 3-Bayesian inference	1-ML unpartitioned analysis 2-ML partitioned analysis	1-ML unpartitioned analysis 2-ML partitioned analysis	1-ML unpartitioned analysis 2-ML partitioned analysis	Bayesian inference
ML analyses: A: Number of partitions B: Number of replicates for bootstrapping convergency C: Final LogLikelihood	1-A: 1, B: 650, C: -644,966.05 2- A: 46, B: 1,400 C: -635,825.11	1-A: 1, B: 400, C: -923,846.29 2- A: 84, B: 300 C: -916,880.02	1-A: 1, B: 1,650, C: -1,994,669.19 2- A: 72, B: 450 C: -1,946,176.72	1-A: 1, B: 1,400, C: -338,081.78 2- A: 11, B: 450 C: -335,384.60	
Bayesian inference: Number of converging runs	2				4

### Assembly of Off-Target Plastome Sequences

Following the approach described by Herrando-Moraira et al. (2019), we assembled plastid genome sequences from the off-target reads using the MIRA v.4.0.2 multi-pass DNA sequence data assembler/mapper (Chevreux et al., 1999) with the MITObim v.1.9 wrappers originally developed for *de novo* assembly of mitochondrial genomes (Hahn et al., 2013). In a three-step process, the reads are mapped by MIRA to a phylogenetically related reference genome (*L. sativa* DQ_383816.1) to identify the more conserved regions between the total readpool and this initial reference and then assembled into contigs, yielding a new and gapped reference sequence. In the second step, the MITObim wrapper script uses the gapped reference to fish in the readpool for partly or fully overlapping reads. In the third step, the overlapping reads are mapped to the gapped reference sequence and incorporated, using MIRA again. The two last steps are iteratively repeated until stationarity of the mapped and assembled reads are reached (Hahn et al., 2013). The assembled plastid genomes were annotated with the web application GeSeq (Tillich et al., 2017), using *Cichorium intybus* (NC_043842.1) and *L. sativa* (NC_007578.1) as references and, after reorganizing the sequence designation in the resulting GeSeq multi-fasta files to start with the sample designation, the Phyluce package (Faircloth, 2015) script “phyluce_assembly_explode_get_fastas_file” was applied for separating the individual coding regions (CDS) into individual gene files. The resulting dataset is hereon referred to as plastome CDS (Table 1 and Figure 2).

### Sequence Alignment, Alignment Trimming, and Summary Statistics

All sequence matrices [COS contigs (OLS and ELS), COS supercontigs, plastome CDS; Table 1 and Figure 2] were aligned individually for each locus or gene, respectively, using MAFFT v.7.266 (Katoh and Standley, 2013) with the parameter –auto. Phyutility vers. 2.2 (Smith and Dunn, 2008) was used to trim alignments by removing sites with a threshold of 50% missing data. Supercontig alignments may represent misassembled contigs, therefore, trimAl v.14 (Capella-Gutiérrez et al., 2009) was used to remove spurious sequences and poorly aligned regions applying the parameters -resoverlap 0.65, -seqoverlap 70, and the gappyout (settings tested in Jones et al., 2019). The same procedure but with the automated1 function was applied to plastome CDS alignments. To avoid obstruction of later tree calculation, locus or gene designations were removed from the sample names in the alignments of the supercontigs and plastid DNA matrices. The tool AMAS (Borowiec, 2016) was then used to retrieve summary statistics for the alignments. Based on these summaries, loci represented in less than 50% of the samples in COS contigs and COS supercontigs alignments were removed. In the plastome CDS alignment 124 samples were represented, for which plastid genomes were successfully assembled. Before the analyses, the plastome CDS alignment was subject to visual inspection and manual cleaning from apparently mis-assembled sequence portions and loci with less than 50% taxa, and less than 10 parsimony-informative (PI) sites were removed from the primary 89 loci dataset, and subsequently, 71 loci were retrieved (plastome CDS71 dataset). For the Bayesian analysis a second, smaller matrix was created from which all genes with less than 50 PI sites (proportion ≤ 0.03%) were removed (plastome CDS26 dataset).

### Phylogenetic Analyses

Phylogenetic reconstructions were conducted for three types of datasets: COS contigs (OLS, ELS), COS supercontigs, and plastome CDS (Table 1 and Figure 2). The sampling of both the COS contigs (160 samples) and plastome CDS datasets (124 samples) was based on selecting the representatives of subtribe Scorzonerinae as ingroup and outside of this subtribe as outgroup. To avoid homology assessment problems in the aligning of the flanking intron sequences as would be expected for remote outgroup members in the COS supercontigs analyses, the early diverging Scorzonerinae genus *Gelasia* was selected as the outgroup for this dataset, and a total of 124 samples across the remainder of the subtribe were included as ingroup (Appendix).

The three types of datasets were analyzed separately under two principal approaches: (1) the multispecies coalescent approach (Liu et al., 2019), in which the species tree is estimated from the individual gene trees resulting from phylogenetic analyses of each locus or gene, and (2) the concatenation approach, using a supermatrix of the concatenated locus or gene alignments for tree inference with ML or Bayesian analyses.

In the multispecies coalescent approach, we first calculated individual gene trees with RAxML-NG using ParGenes (Morel et al., 2019), as a tool for parallel model testing and tree inference of numerous individual loci or genes. ParGenes uses Modeltest-NG (Darriba et al., 2019) to calculate the scores for the best model; we selected BIC as the model test criterium. Parameters for RAxML-NG were set as follows: 50 ML tree searches using 25 parsimony-starting-trees + 25 random-starting-trees; bootstrapping either with the autoMRE option in effect (disables multinodal parallelization of runs), a requested maximum of 3,000 replicates and using the default threshold of 0.03 for the BS convergence assessment (COS contigs and supercontigs datasets), or without autoMRE option and 1,200 replicates requested (plastome CDS dataset); mapping the BS support values onto the best-scoring/best-known ML tree. Recent studies have questioned the traditional assumption of the plastome as a uniform and single locus, indicating that it is rather a mosaic of genes evolving under different constraints (Gonçalves et al., 2019; Walker et al., 2019). Therefore, we also applied the multispecies coalescent approach to the plastome CDS dataset to compare phylogenetic inference with the concatenation approach. Following the recommendations by Mirarab (2019), Newick Utilities (Junier and Zdobnov, 2010) were used before the species’ tree calculation with ASTRAL vers. 5.6.3 (Mirarab and Warnow, 2015), to collapse nodes with very low support (less than 10%), and TreeShrink v. 1.3.1 (Mai and Mirarab, 2018) with standard parameters was used to detect outliers with abnormally long branches and to remove such samples from individual gene trees. ASTRAL was then used to generate the species tree by maximizing the number of quartet trees shared between gene trees and the species tree (Mirarab and Warnow, 2015) and calculating local posterior probabilities (LPP; Sayyari and Mirarab, 2016) as branch support values. The nodes with LPP < 0.5 were considered as not statistically supported and were collapsed in all coalescent species trees. The levels of discordance between the individual nuclear gene trees were assessed with the program PhyParts (Smith et al., 2015). The ELS ASTRAL species tree was used as a reference tree. PhyParts requires rooted trees with the same outgroup. Therefore, 190 individual gene trees and the species tree of the ELS dataset were rooted with *C. arenaria* as an outgroup using R and the package APE (Paradis and Schliep, 2019) after 19 gene trees missing *C. arenaria* were excluded. With the script phypartspiecharts.py (Johnson, 2017), the output of PhyParts was visualized by plotting pie charts on the reference tree that show the proportions of concordant and discordant gene trees for each bipartition.

In the concatenation approach, the alignments of each type of dataset were combined into a supermatrix using AMAS (Borowiec, 2016). ML analyses were run using the Multi-Point Interface (MPI) version of RAxML-NG v. 0.8.1 and 0.9.0 (Kozlov et al., 2019). To assess the significance of the partitioning and choice of DNA substitution models for our datasets, two analyses were run: one with non-partitioned datasets and the other with partitioned datasets. The general GTR+G model of sequence evolution was applied to the non-partitioned concatenated datasets. For the partitioned dataset, the best partitioning schemes and substitution models were obtained using PartitionFinder v.2 (Lanfear et al., 2017) with the relaxed clustering algorithm criterion as recommended by Lanfear et al. (2014) for large phylogenomic datasets with the “rcluster” search option (–rcluster-max 100 and –rcluster-percent 0.1), “BIC” model selection parameters, linked branch lengths, and a choice between three substitution models (GTR, GTR+G, and GTR+I+G). Using RAxML-NG, the tree space was explored with 50 (for the plastome CDS71 and COS contigs datasets) or 20 (COS supercontigs datasets) ML tree searches using 25 or 10 random and 25 or 10 parsimony-based starting trees, respectively, followed by standard bootstrapping, which employed the bootstopping test with a maximum replicate number and a bootstrap (BS) convergence requirement with 3% default cutoff for each dataset (Table 1). The BS support values were mapped onto the best-scoring ML tree obtained and the nodes with BS of <50% were collapsed in all ML concatenated trees.

Because the phylogenetic reconstructions of both the gene trees (as input for the multispecies coalescent analyses) and the species trees based on the concatenated matrix were conducted with ML using RAxML-NG, and tree inference with RAxML-NG can occasionally be misled by non-randomly distributed missing data (Xi et al., 2015), we also applied a Bayesian analysis on the concatenated matrices to test the robustness of the reconstructions. We used PhyloBayes (Lartillot et al., 2013; Lartillot, 2020), which implements a non-parametric approach based on Dirichlet process priors to model nucleotide or amino acid substitutions as site-specific random variables directly inferred from the data, as opposed to being specified *a priori*. This model classifies amino acid or nucleotide sites into topological categories (therefore “CAT model”). We applied the default CAT-GTR and a discretized gamma distribution with four categories, which are computationally resource-intensive but have shown to perform well in inferring accurate branching patterns in genomic datasets (Whelan and Halanych, 2017; Lartillot et al., 2018). Convergence of the chains can become challenging for larger alignments beyond 20,000 sites (Lartillot et al., 2018), therefore, six chains were independently run for >25,000 cycles, and their stationarity, appropriate burn-in, and convergence were first visually assessed using Tracer vers. 1.7.1 (Rambaut et al., 2018). Subsequently, convergence in tree space and reproducibility of the posterior consensus trees across chains was assessed using the bpcomp and tracecomp commands of PhyloBayes. Chains with an insufficient sampling of parameters [low ESS (=effective sampling size)] and chains stuck in a local optimum (lower loglikelihood values) and, thus, badly converging with the others were discarded. A minimum of two well-converging chains with the highest loglikelihood values was used to calculate the final posterior consensus trees.

The COS contigs and plastome CDS species trees were rooted with the members of the Scolyminae clade (*S. hispanicus* and *C. arenaria*) because this is the earliest diverging clade of the tribe in our sampling (Kilian et al., 2009a; Tremetsberger et al., 2012). The COS supercontig tree with its more restricted sampling was rooted with the *Gelasia* clade (Appendix). TreeGraph vers. 2 (Stöver and Müller, 2010), was used for viewing and displaying the phylogenetic trees. Before, format conversion in FigTree vers. 1.4.4. (Rambaut, 2018) was necessary in some cases.

Addressing the unusual diversity of pollen types in the subtribe as an exemplar, we wanted to test if the resolved phylogenetic relationships help to explain the current distribution of morphological character states in the subtribe. To reconstruct the pollen types at ancestral nodes, we used the backbone of the ELS ASTRAL tree as a phylogenetic hypothesis and treated the seven pollen types identified by Blackmore (1982) as unordered states. The matrix was built and the parsimony ancestral characters state reconstruction was done with Mesquite vers. 3.7 (Maddison and Maddison, 2014; Mesquite Project Team, 2014).

## Results

### Hyb-Seq Data Processing and Loci Assemblies

In total, 163 samples were analyzed in our study, including both nuclear and plastome datasets, and for 157 of them, Hyb-Seq data were newly created (Appendix). The DNA was isolated in most cases directly from herbarium specimens of the following age ranges: 3 collected from ≤ 1900, 12 from > 1900 ≤ 1950, 65 from > 1950 ≤ 2000 and 77 (12 of which from silica-dried material) from > 2000.

The average number of reads per sample obtained was 4,952,940, ranging from 147,379 in *Scorzonera laciniata* DB 44268 to 25,313,374 in *H. achyrophorus* DB 546. On average, the percentage of mapped reads per sample was 35% (range: 7–58%) and the number of COS loci recovered for each sample ranged from 423 in *S. laciniata* DB 44268 to 1,041 in *Pseudopodospermum picridioides* DB 44329, with a mean of 968 loci of the total of 1,061 target loci in the set. A total of 897 COS loci and 832 COS supercontig loci were identified by HybPiper as loci containing potentially paralog sequences and removed from the datasets before analyses. After removing the spurious sequences of the matrices in the COS supercontigs dataset in trimAl and eliminating the loci with less than 50% taxa in both datasets, the OLS dataset and COS supercontigs datasets yielded 147 and 148 loci, respectively. The final concatenated OLS dataset had a length of 44,634 bp, of which 16,315 were parsimony informative (∼35%) and an average of 8% missing data per locus (range: 0–27.5%; Table 1). The final concatenated sequences of the COS supercontigs dataset had a length of 132,646 bp, of which 65,023 were parsimony informative (∼48%) and an average of 7.5% missing data per locus (range: 3.9–11.1%; Table 1). Visual inspection of the 897 gene trees of loci with paralog warnings, suggested that the multiple sequences of 70 loci represented allelic variants, not paralogs. Apart from six loci containing less than 50% taxa, these loci were added to the OLS dataset, forming the enlarged ELS dataset of 211 loci. The final concatenated sequences of the ELS dataset had a length of 64,358 bp, consisting of 23,379 parsimony informative sites (∼35%) and an average of 8.6% missing data per locus (range: 0–27.4%; Table 1). The statistics for the OLS, ELS, and supercontigs datasets before (Supplementary Table 1) and after (Table 1) removing the loci with less than 50% taxa revealed a slight increase in the percentage of variable and PI sites in the condensed datasets, but with no considerable impact on the average percent of missing data per locus. Reducing the number of loci in the plastome dataset from the original 89 to 71 by eliminating those with less than 50% taxa and less than 10 PI sites (plastome CDS71 dataset) caused a reduction in the percentage of variable and PI sites, as the well as the length of concatenated alignments and average sequence length per locus (Supplementary Table 1 vs. Table 1). The concatenated plastome CDS71 sequence was 63,917 bp long, containing 6,321 PI sites (∼8%), and an average of 25.9% missing data per locus (6.6–47.4%). The plastome CDS26 dataset built by removal of all genes with less than 50 PI sites had 26 genes, the concatenated sequence had a length of 48,478 bp, containing 5,300 PI sites (∼11%), and an average of 25% missing data per locus (10–38.8%; Table 1, plastome CDS26 dataset).

### Phylogenetic Inference

**FIGURE 3:**
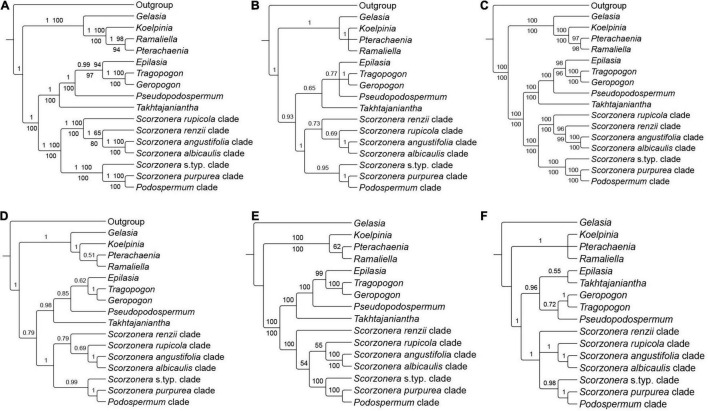
Backbone topology of phylogenetic relationships among major lineages of subtribe Scorzonerinae, inferred from different nuclear [original loci set (OLS), enlarged loci set (ELS), supercontig] datasets under the concatenation [Maximum Likelihood (ML) analysis performed on no-partitioned and partitioned concatenated datasets and Bayesian inference] and coalescent approaches. **(A)** OLS concatenated species tree, posterior probabilities (PP) from Bayesian analysis and bootstrap values from ML no-partitioned analysis (BS-NP) above the branches and bootstrap values of ML partitioned analysis (BS-P) below the branches; **(B)** OLS coalescent species tree; **(C)** ELS concatenated species tree, BS-NP support values of ML no-partitioned analysis above the branches and BS-P of ML partitioned analysis below the branches; **(D)** ELS coalescent species tree; **(E)** Supercontig concatenated species tree, BS-NP support values of ML no-partitioned analysis above the branches and BS-P of ML partitioned analysis below the branches; and **(F)** Supercontig coalescent species tree. Branch labels of coalescent species trees are corresponded to local posterior probabilities (LPP). Branches with less than 50% bootstraps (BS-NP and BS-P), 0.5 PP and 0.5 LPP are collapsed.

In the current study, fourteen phylogenetic analyses based on the nuclear (OLS, ELS, and supercontigs) and plastome CDS datasets were performed under ML and Bayesian inference based on concatenated loci and under the multispecies coalescent model based on the individual gene trees (Table 1; Figures 2, 5–7; and Supplementary Figures 1–5). Trees estimated under the multispecies coalescent model resulted in a lower mean of support values and percentages of nodes with maximum support than those obtained under the concatenation approaches (Table 2). No hard topological incongruences exist between the species trees of both principal approaches (coalescent and concatenation), apart from the single contrary consecutive sister group relationship of the monospecific *Scorzonera renzii* and *S. rupicola* clades (Figure 3). Collapsing nodes with very low support and removing outlier samples with abnormally long branches from the gene trees using TreeShrink, before running coalescent analyses, did not cause any notable difference in topology and support values, compared to trees without employing TreeShrink (not shown).

**TABLE 2:** A summary of “Mean of support values” and “Percentage of nodes with full support (100% BS, 1 PP, and 1 LPP)” across all analyses.

Dataset/analysis	Mean support value	Percentage of nodes with full support (100% BS, 1 PP, 1 LPP)
OLS/ML unpartitioned analysis	87.2%	41.6
OLS/ML partitioned analysis	89.9%	43.5
ELS/ML unpartitioned analysis	89.9%	45.4
ELS/ML partitioned analysis	89.6%	46.5
COS supercontig/ML unpartitioned analysis	88.2%	41.4
COS supercontig/ML partitioned analysis	90.3%	44.5
Plastome CDS71/ML unpartitioned analysis	86.1%	31.1
Plastome CDS71/ML partitioned analysis	85.9%	31.5
OLS/coalescent approach	0.84 PP	37.8
ELS/coalescent approach	0.86 PP	41.8
Supercontig/coalescent approach	0.88 PP	41
Plastome CDS/coalescent approach	0.81 PP	31
OLS/Bayesian inference	0.95 PP	71.3
Plastome CDS26/Bayesian inference	0.89 PP	42.4

**FIGURE 4:**
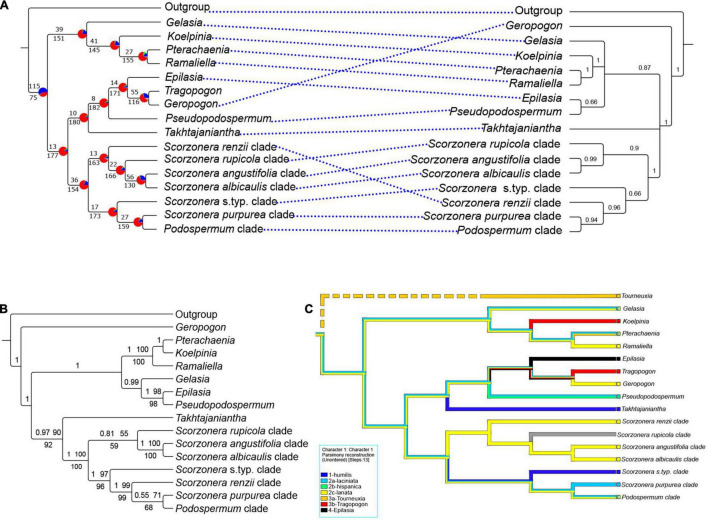
**(A)** Mirrored nuclear ELS coalescent backbone (left) and plastome CDS coalescent trees (right); the ELS tree with a summary of concordant and discordant gene trees. For each branch, the top number indicates the number of concordant gene trees and the bottom numbers those of conflicting gene trees. The pie charts indicate the proportion of gene trees that support that clade (blue), the proportion that supports the main alternative for that clade (green), the proportion that supports all other topologies (red) or the proportion of uninformative gene trees for that clade (gray); the plastome CDS tree with local posterior probability (LPP) support values. **(B)** Backbone topology of plastome CDS concatenated species tree, posterior probabilities (PP) of Bayesian analysis and bootstrap values of ML no-partitioned analysis (BS-NP) above the branches and bootstrap values of ML partitioned analysis (BS-P) below the branches. **(C)** Parsimony ancestral character state reconstruction on the nuclear ELS coalescent backbone tree for the seven pollen types of the Scorzonerinae identified by Blackmore (1982). The numerals in the state designations indicate the four hypothetical principal evolutionary lines of pollen types and the letters the subordinate lines according to Blackmore (1982; Figure 7). *Tourneuxia* is included as a putative early diverging lineage (indicated by the broken line) following Zaika et al. (2020). Except in the ELS tree of A, branches with less than 50% bootstraps (BS-NP and BS-P), 0.5 PP and 0.5 LPP are collapsed.

**FIGURE 5:**
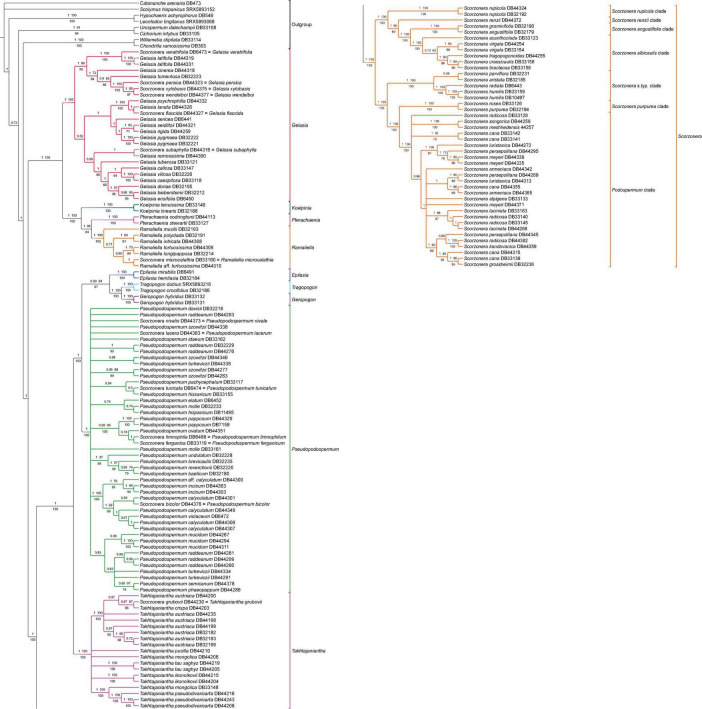
Phylogenetic reconstruction of subtribe Scorzonerinae inferred with OLS dataset under the concatenation approach. Branch labels above the branches indicate posterior probabilities (PP) of Bayesian analysis and bootstrap values of ML no-partitioned analysis (BS-NP) and below the branches, bootstrap values from ML partitioned analysis (BS-P). Branches with less than 0.5 PP and 50% bootstraps (BS-NP and BS-P) are collapsed.

**FIGURE 6:**
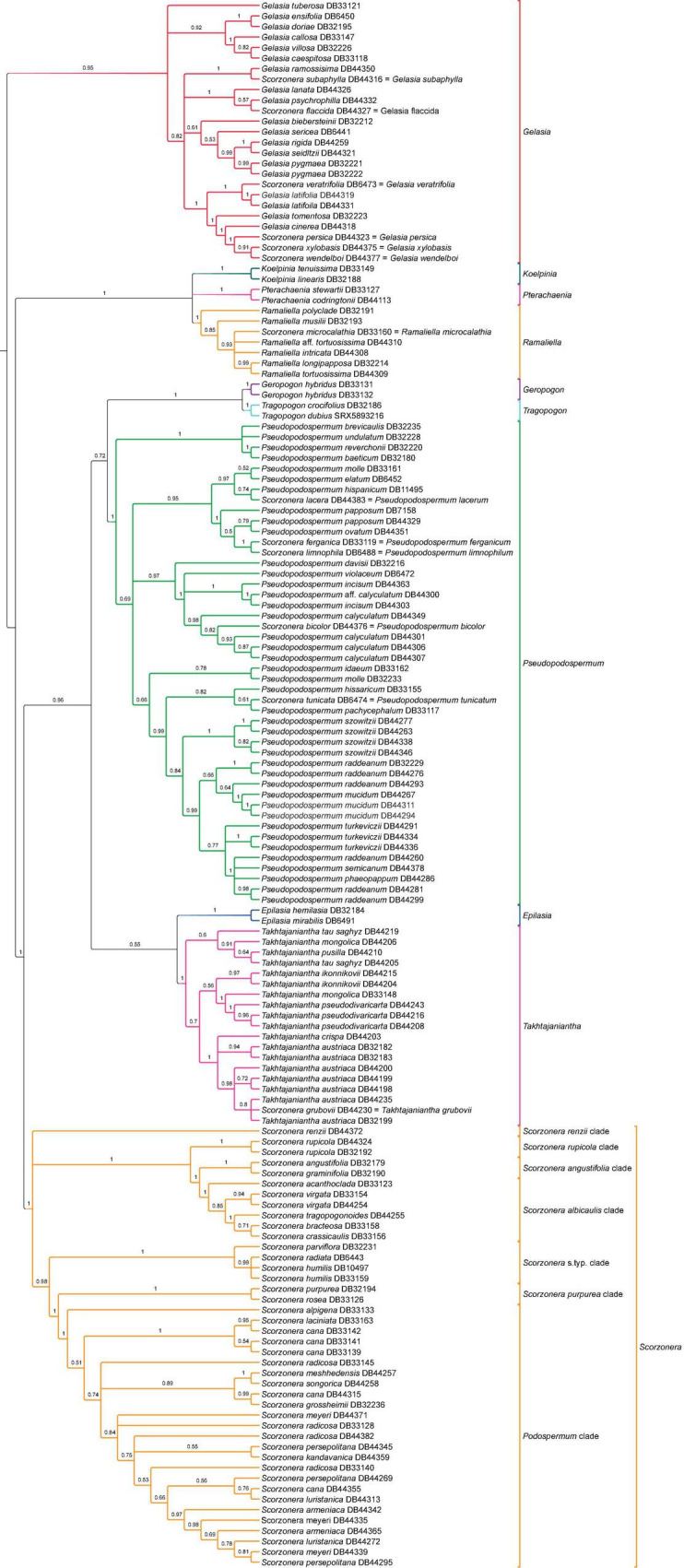
Coalescent species tree of subtribe Scorzonerinae based on the supercontig dataset. Branch labels indicate support values of local posterior probabilities (LPP) and branches with less than 0.5 LPP are collapsed.

**FIGURE 7:**
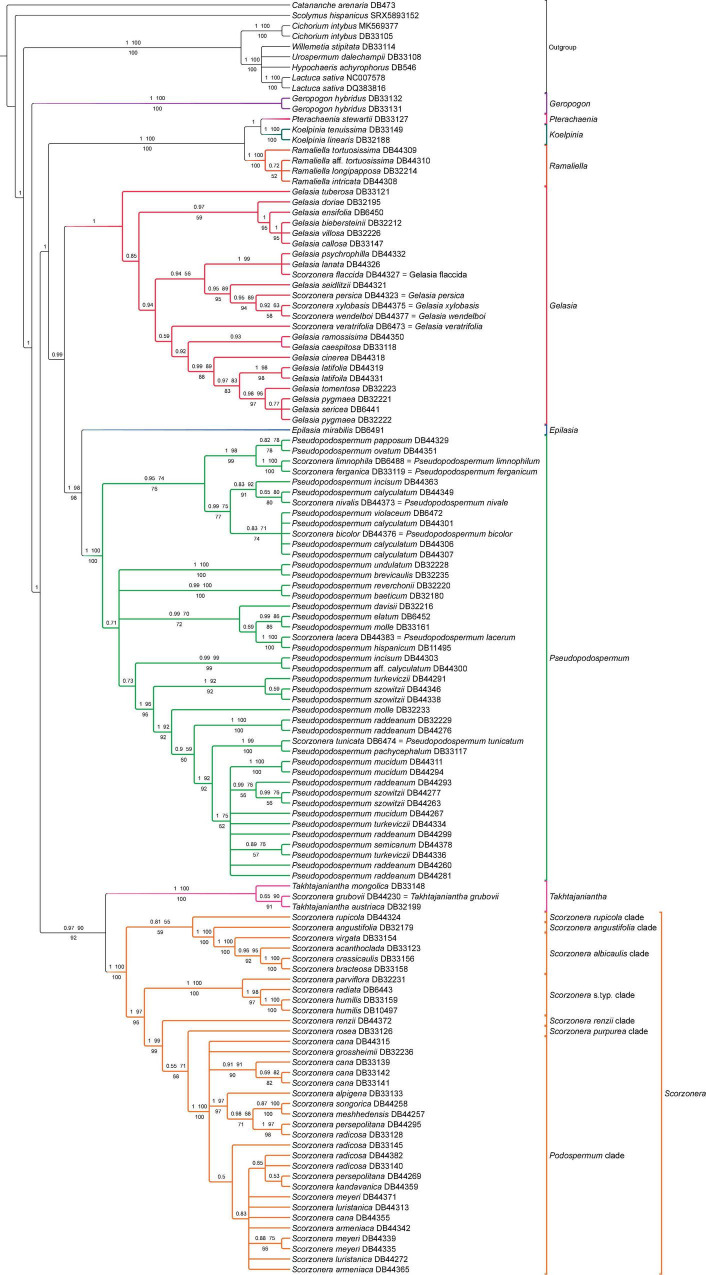
Phylogenetic reconstruction of subtribe Scorzonerinae inferred with plastome CDS dataset under concatenation approach. Branch labels above the branches indicate posterior probabilities (PP) of Bayesian analysis and bootstrap values of ML no-partitioned analysis (BS-NP) and below the branches, bootstrap values from ML partitioned analysis (BS-P). Branches with less than 0.5 PP and 50% bootstraps (BS-NP and BS-P) are collapsed.

In general, deep tree branches were well-supported and the backbone topology was largely congruent in all inferences of each dataset (Figures 3, 4), while the internal resolution of the lineages was comparatively poor for all datasets (Figures 5–7). However, some small and large groups with congruent topologies and strong support values were also found within major lineages across the trees, and reciprocally, the corresponding tips of some well-supported subclades in shallow nodes generated most cases of topological incongruences between different analyses (Figures 5, 7).

Technical details regarding phylogenetic analyses conducted on the COS contigs (OLS and ELS), COS supercontigs, and plastome CDS datasets are given in Table 1. Hereon the support values are provided in parenthesis in the following order: ML analysis on the unpartitioned concatenated dataset [bootstrap percentage (BS-NP)], ML analysis of partitioned concatenated dataset [bootstrap percentage (BS-P)], Bayesian inference [posterior probability (PP)], and coalescent-based approach (LPP).

Overall, the phylogenetic inferences in this study based on the nuclear on-target and plastome off-target Hyb-Seq data revealed the same major clades as those inferred by Zaika et al. (2020). Clade designations, therefore, follow Zaika et al. (2020: Figures 1, 2).

The exemplary ancestral character state reconstruction for the seven pollen types on the nuclear backbone tree is provided in Figure 4C and Supplementary Data Sheet 1. The distribution of the pollen types in the lineages of the Scorzonerinae is characterized by multiple state dimorphisms and the reconstruction at ancestral notes shows many homoplasies.

### Nuclear Tree Inferences

#### Conserved Ortholog Set Contig Tree Inferences

All inferred phylogenies conducted on the COS contigs datasets (OLS and ELS) confirmed the monophyly of subtribe Scorzonerinae and resolved the same major phylogenetic lineages of the subtribe as identified by Zaika et al. (2020: Figure 1) with maximum statistical support (Figures 3, 5 and Supplementary Figures 1–3). These are *Epilasia*, *Gelasia*, *Geropogon*, *Koelpinia*, *Pseudopodospermum*, *Pterachaenia*, *Ramaliella*, *Scorzonera*, *Takhtajaniantha*, and *Tragopogon*. Also, within *Scorzonera*, the same seven clades were resolved with full support (Figure 3) as in Zaika et al. (2020: Figure 1). Among these lineages and clades, *Geropogon*, *Scorzonera rupicola*, and *S. renzii* are monospecific.

Regarding the relationships between the major clades, the backbone topology of the coalescent-based analyses shows single not statistically supported nodes (OLS coalescent species tree) or low support values for single clades (ELS coalescent species tree), whereas always fully resolved nodes and fully or strongly supported clades in the concatenation approach (Figure 3). The 30% increase in the number of loci in the ELS dataset led to a moderate increase in support values of the partitioned and unpartitioned analyses of the ELS in the phylogenetic backbone compared to these two analyses of the OLS (Figures 3A,C). The increase in support and resolution within the major clades was somewhat more significant. For instance, the clade consisting of the *S. albicaulis* and *S. angustifolia* clades was resolved as sister to the *S. renzii* clade in ML-concatenation analyses of ELS with high support (96 BS-NP, 99 BS-P), whereas with distinctly lower support in those of OLS (65 BS-NP, 80 BS-P). Concerning the shallower nodes, the support for the sister group relationship of *Ramaliella longipapposa* DB 32214, and *Ramaliella tortuosissima* DB 44309 was improved in ML concatenation analyses of the ELS (BS-NP 97, BS-P 97), compared to both ML concatenation analyses of the OLS (70 BS-NP, 70 BS-P), see Figure 5 vs. Supplementary Figure 1. In all COS contigs inferences (Figures 3A–D), the subtribe Scorzonerinae was split into two well-supported clades, one composed of *Gelasia*, *Koelpinia*, *Pterachaenia*, and *Ramaliella* (full support), and the others of *Epilasia*, *Geropogon*, *Pseudopodospermum*, *Scorzonera*, *Takhtajaniantha*, and *Tragopogon* (OLS: 100 BS-NP, 100 BS-P, 1 PP, 0.93 LPP; ELS: full support). In the first clade, *Gelasia* was resolved as sister to a clade in which *Ramaliella* was sister to *Pterachaenia* (OLS: 98 BS-NP, 94 BS-P, 1 PP; ELS: 97 BS, 98 BS), and these together as sisters to *Koelpinia* (full support) based on ML and Bayesian concatenated analyses (Figures 3A,C). The backbone of the coalescent analysis differed in that the clade including *Koelpinia*, *Pterachaenia*, and *Ramaliella* was internally not statistically supported (Figures 3B,D). In the second clade, *Scorzonera* was resolved as sister to the clade of the remaining lineages in which *Takhtajaniantha* was inferred as sister to a clade comprising *Epilasia*, *Geropogon*, *Pseudopodospermum*, and *Tragopogon* with full support in concatenated analyses while not supported in coalescent analysis (0.65 LPP).

In all analyses of the concatenated matrix (Figures 3A,C), a fully supported clade, comprising *Geropogon* and *Tragopogon*, was resolved as sister to *Epilasia* (OLS: 94 BS-NP, 97 BS-P, 0.99 PP; ELS: 98 BS-NP, 96 BS-P) and this clade in turn as sister to *Pseudopodospermum* (OLS: 98 BS-NP, 100 BS-P, 1 PP; ELS: 100 BS-NP, 100 BS-P). In the coalescent analysis, the relationships between these three clades were unresolved, forming a polytomic structure (Figures 3B,D).

Within the *Scorzonera* clade, the analyses of the concatenated matrix resolved two well-supported clades (Figures 3A,C): in the one clade, the *Podospermum* clade was sister to the *S. purpurea* clade, and both, in turn, sister to the *Scorzonera* s.typ. clade with maximum support. In the other clade, the fully supported *S. albicaulis* and *S. angustifolia* clades were sisters to the *S. renzii* clade (OLS: 65 BS-NP, 80 BS-P, 1 PP; ELS: 96 BS-NP, 99 BS-P) and they, in turn, were sister to the *S. rupicola* clade with full support. The coalescent analysis revealed the same topology, however, with lower support for some clades (Figures 3B,D).

Analysis of the discordance between the individual ELS gene trees with PhyParts (Figure 4A for the backbone tree, Supplementary Figure 6 for the full tree) shows that the root of the Scorzonerinae was supported by 115 (60%, blue portion of the pie chart, Figure 4A) of all gene trees. In contrast, within the Scorzonerinae, there was a high level of discordance. In the backbone tree, only a minority of gene trees (4–30%, Figure 4A) support the nodes, and the full tree (Supplementary Figure 6) shows a similar picture.

#### Conserved Ortholog Set **S**upercontigs **T**ree **I**nferences

Analyses of the supercontig data matrices using ASTRAL and concatenated ML approaches (unpartitioned and partitioned data matrices) resulted in largely similar topologies for the phylogenetic backbone of the Scorzonerinae. The backbone nodes were well-supported in the concatenated analyses, while the multispecies coalescent analysis revealed some nodes that were not statistically supported and otherwise had lower nodal support (Figures 3E,F). A fully supported clade comprising *Koelpinia*, *Pterachaenia*, and *Ramaliella*, was resolved with full support as sister to the rest of the ingroup. The coalescent approach retrieved *Pterachaenia*, *Ramaliella*, and *Koelpinia* in a polytomic structure and the topologies obtained under concatenation analyses revealed low support for a sister relationship between *Pterachaenia* and *Ramaliella* (62 BS-NP) or between *Koelpinia* and *Ramaliella* (72 BS-P). A clade including the remainder of the subtribe was split into two well-supported clades: one comprising *Epilasia*, *Geropogon*, *Pseudopodospermum*, *Takhtajaniantha*, and *Tragopogon* (100 BS-NP, 100 BS-P, 0.96 LPP), the other the *Scorzonera* lineage. The same topology was revealed for the relationships of these lineages in the concatenated and coalescent trees, however, the nodes had lower support in the coalescent analysis. Within the *Scorzonera* lineage, the concatenation and coalescent inferences showed congruence in the backbone topology of major clades including the *Podospermum* clade, *S. purpurea* clade, *Scorzonera* s.typ. clade, *S. albicaulis* clade, and *S. angustifolia* clade, but different positions of the *S. renzii* and *S. rupicola* clades across trees.

In contrast to the nearly complete backbone congruence between the various analyses of the nuclear datasets, the corresponding tips in shallow nodes generated most cases of topological incongruences and nodal support differences for species relationships (Figures 5, 6 and Supplementary Figures 1–4). We will give these full trees closer consideration in the section “Discussion.”

### Plastome Off-Target Inferences

The phylogenetic backbones of the four analyses based on the plastome CDS matrix were largely congruent with each other (Figures 4, 7 and Supplementary Figure 5). Analyses of the plastome dataset resolved the same major lineages as in the analyses of the nuclear (COS contigs and COS supercontigs) datasets, but their relationships showed several conspicuous differences (Figures 3, 4). This concerns the relationships of *Gelasia*, *Geropogon*, *Takhtajaniantha*, the *S. rupicola*, and *S. renzii* clades, and the clade comprising *Koelpinia*, *Pterachaenia*, and *Ramaliella*.

Subtribe Scorzonerinae was resolved as monophyletic with full support. Within the subtribe, *Geropogon* was resolved as sister to the remainder of the subtribe, also with full support in all analyses; *Tragopogon* was not represented in the plastome datasets. ML and Bayesian concatenated analyses inferred a sister relationship between a clade containing *Takhtajaniantha* as sister to *Scorzonera* (90 BS-NP, 92 BS-P, 0.97 PP) and another clade including *Epilasia*, *Gelasia*, *Koelpinia*, *Pseudopodospermum*, *Pterachaenia*, and *Ramaliella* (99 BS-NP, 99 BS-P, 1 PP), while in the coalescent analysis, the relationship between *Takhtajaniantha* and the *Scorzonera* lineage was not statistically supported and the clade, therefore, collapsed, resulting in a trichotomy (Figures 4A,B). *Koelpinia, Ramaliella*, and *Pterachaenia* formed a clade with full support in all analyses, while their internal relationship was not statistically supported by the ML trees and incongruent in the other two (Figures 4A,B). *Gelasia, Epilasia*, and *Pseudopodospermum* formed a second clade, which was supported only in the Bayesian analysis (0.99 PP), with *Gelasia* as sister to *Epilasia* and *Pseudopodospermum*; in the ML and Bayesian trees, the sister group relationship between *Epilasia* and *Pseudopodospermum* was resolved (98 BS-NP, 98 BS-P, 1 PP), while in the ASTRAL tree, this relationship was not statistically supported (Figures 4A,B). Within the *Scorzonera* lineage, all inferences indicated similar backbone relationships among major clades. A sister-group relationship between the *S. angustifolia* and *S. albicaulis* clades was supported in all analyses (100 BS-NP, 100 BS-P, 1 PP, 0.99 LPP). The relationship of the *Scorzonera* s.typ. clade, as sister to the clade including the *Podospermum, S. purpurea*, and *S. renzii* clades, was resolved in both the Bayesian and ML trees (99 BS-NP, 96 BS-P, 1 PP) but not statistically supported in the ASTRAL tree, and the relationship of the *S. renzii* clade, as sister to the clade formed by *Podospermum* and the *S. purpurea* clades was resolved in all analyses (99 BS-NP, 99 BS-P, 1 P, 0.96 LPP). However, the sister relationship of *Podospermum* and the *S. purpurea* clade was recovered with low support (71 BS-NP, 68 BS-P, 0.55 P, 0.94 LPP; Figures 4A,B).

Similar to the results for the nuclear datasets, congruence in the phylogenetic backbones of the different analyses of the plastome datasets contrasts with frequent cases of topological incongruences and differences in statistical support values in shallow nodes (Figure 7 and Supplementary Figure 5). A closer consideration follows in the section “Discussion.”

## Discussion

### Impacts of Hyb-Seq Datasets and Approaches on the Robustness of Phylogenetic Inference

Our study presents the first phylogenomic analysis of the Scorzonerinae based on the myBaits COS Compositae 1Kv1 (Mandel et al., 2014). Most of the data were generated from herbarium specimens; 38 (24%) of the samples newly sequenced were 50 years or older, which demonstrates the power of Hyb-Seq in the valorization of the wealth of material preserved in herbaria around the world.

In principle, species trees estimated under the multispecies coalescent model and trees from the concatenation approach were congruent. However, there were lower means of support values and percentages of nodes with maximum support in coalescent model trees compared with those from the concatenated approach. Removal of outlier samples with abnormally long branches from the gene trees with TreeShrink did not cause any notable difference in topology and support values of the coalescent model trees. This indicates that our datasets were largely free from significant biases caused by contamination, mistaken orthology, and misalignment, which are common reasons for long branches (Mai and Mirarab, 2018). Low support values and statistically unsupported nodes in species trees of the multispecies coalescent approach are due to a lack of support in individual gene trees or gene tree discordance. The PhyParts output (Figure 4A) demonstrates massive discordance among the gene trees in the Scorzonerinae in that all backbone nodes were supported only by a minority of gene trees and the vast majority of shallower nodes have similar levels of discordance (Supplementary Figure 6). In contrast, the root of the Scorzonerinae was found in 60% of the gene trees, markedly corroborating them as a separate lineage. Important sources of gene tree discordance are hybridization and incomplete lineage sorting (Liu et al., 2015) because of ancient rapid radiation (Whitfield and Lockhart, 2007; Oliver, 2013). Trees based on the multispecies coalescent approach better reflect the phylogeny than such based on concatenated matrices, because the multispecies coalescent model takes incomplete lineage sorting into account in that it allows conflicting phylogenetic signals among individual loci and provides a more realistic measure of support (Liu et al., 2015, 2019; Sayyari and Mirarab, 2016). We, therefore, conclude that other biological causes such as reticulate or convergent evolution (Liu et al., 2019) remain the main causes of gene tree discordance in the Scorzonerinae.

Among the analyses of concatenated datasets, partitioning led to a slightly higher percentage of nodes with full support and typically a higher mean of the support values, compared to the trees estimated from unpartitioned datasets applying the generic nucleotide substitution model GTRGAMMA (Table 2). Log-likelihood scores were also higher for ML trees with partitioning (Table 1). Our findings, thus, confirmed the positive impact of data partitioning on tree resolution and support values, concordant with previous studies (Xi et al., 2012; Kainer and Lanfear, 2015; Warnow, 2015; Lanfear et al., 2017; Jones et al., 2019), although the impact is not striking in our case.

Adding flanking introns of the COS exons to generate supercontig datasets increased the alignment length nearly threefold, the number of variable and PI sites more than three- and four-fold, respectively, and correspondingly, the percentage of PI sites increased (c. 37%). This was expected because the non-coding intron regions possess a great amount of variation and are less sensitive to selective pressures, such as selection-driven convergence (Weitemier et al., 2014; Folk et al., 2015; Johnson et al., 2016; Gernandt et al., 2018; Kates et al., 2018). The resulting increase in the mean of support values and percentage of nodes with full support in terms of LPP in ASTRAL species trees and BP support levels in ML concatenated analyses, compared to OLS trees, was, nevertheless, altogether moderate but more visible in the species trees of the multispecies coalescent analyses (Table 2). The corresponding full trees (Figures 5–7; Supplementary Figures 1–5) show increased support for branches at deeper nodes and otherwise in particular an improved resolution and support for branches at shallow nodes, leading, in several cases, to a substantial improvement in the reconstruction of species relationships. This agrees with previous studies that highlighted the potential of supercontigs for improved statistical support (Jones et al., 2019; Bagley et al., 2020; Gardner et al., 2021). Signal saturation at most of the deeper nodes and the opposing impact of discordant phylogenetic signals due to reticulate evolution at deeper and, in particular, at shallow nodes may explain the rather moderate impact of supercontigs on support values.

Of the 1,061 COS loci, a significant number were flagged as potentially paralogous by HybPiper (897 COS contigs and 832 COS supercontigs). Jones et al. (2019) discussed the relationship between whole-genome duplications (WGDs) in the evolution of a lineage and the number of paralogous loci. WGDs have played a major role also in the diversification of the Compositae, and the Cichorieae are among the tribes, which are known to have experienced WGDs (Huang et al., 2016). Jones et al. (2019) found 721 loci flagged as paralogous in a small sampling across the tribe, which was the highest value among all Compositae tribes included. At the same time, they reported distinctly smaller numbers for individual subsets of this sampling (Jones et al., 2019: Table 1), confirming that a wider systematic range of a sampling potentially increases the number of putative paralogs. The Vernonieae, for which no WGDs, so far, have been identified, had, however, the second-highest number of putative paralogs, and Jones et al. (2019) assumed that this is due to lineage-specific or even species-specific WGDs within the Vernonieae. The Scorzonerinae may be similar in this respect: the number of putative paralogous loci for the Scorzonerinae here is even higher than in Jones et al. (2019). Moreover, in our study, the number of putative paralogs detected was only slightly increased (6.6%) when comparing the Scorzonerinae only vs. Scorzonerinae plus eight samples from more distantly related Compositae subtribes (supercontigs sampling vs. COS contigs sampling). Therefore, the Scorzonerinae alone appear to be a major source of potential paralogs. WGD, as allo- or autopolyploidy is not apparent in the subtribe with an only exceptional occurrence of polyploidy in single species of *Koelpinia* and *Takhtajaniantha* with a basic number of *x* = 6 or 7 (Zaika et al., 2020). However, lineage diversification and evolution of innovations are not directly initiated by WGD but through processes of post-WGD genome rearrangements, including massive loss of duplicated genes, resulting in post-polyploid genome diploidization (Mandákova and Lysak, 2018). Therefore, the occurrence of WGD in the deeper history of a lineage is rarely manifested by persisting polyploidy. We suggest that the high paralogy found within Scorzonerinae may be evidence for extensive reticulate evolution at different evolutionary timescales.

The extension of our original COS dataset to include loci with paralog warnings that were found to be based on allelic variation only, allowed us to investigate the impact of an increase from 147 loci (in the original set, OLS) by 64 loci or c. 43% to 211 loci (in the extended data set ELS) on the phylogenetic reconstruction. The higher number of loci brought about a corresponding increase in variable and PI sites, whereas their percentages remained almost constant (Table 1). ML concatenation analyses of the ELS dataset resulted in a somewhat improved resolution, indicated by the slightly higher percentage of nodes with full support whereas the topologies obtained from both datasets were largely congruent with each other (Table 2 and Figures 3, 4). Also, in the coalescent analyses, the ELS tree shows improved resolution and support, notably in the case of the successive sister group relationships of *Pseudopodospermum, Takhtajaniantha*, and *Scorzonera* to the *Tragopogon-Geropogon-Epilasia* clade.

### Significance of Phylogenetic Inference From the Plastome and Methodological Considerations

The first plastid DNA phylogeny of the Scorzonerinae by Zaika et al. (2020: Figure 2) confirmed the major lineages found in their nrITS phylogeny that was also corroborated by our analyses but failed to resolve relationships between them (with a few exceptions). Most of the relationships inferred from our plastome CDS dataset are therefore new results and show that cytonuclear discordance (Lee-Yaw et al., 2019) is a major issue already in the backbone of the species trees (Figure 4, discussed in more detail below).

The success in using the off-target reads for assembling plastid genomes has been shown to depend significantly on the spiking of the post-capture library with the unenriched pre-capture library (Jones et al., 2019). Among the 124 samples, of which we could successfully assemble the plastome and build the plastome CDS matrix, more than half were spiked with the unenriched pre-capture library. Considering the effort to assemble and annotate complete plastomes in relation to the cost of a sequencing job, it seems questionable if spiking is a recommendable strategy compared to sequencing unenriched libraries alone. MITObim v.1.9 wrappers (Hahn et al., 2013) for plastome assembly, which makes use of the MIRA v.4.0.2 multi-pass DNA sequence data assembler/mapper (Chevreux et al., 1999) in an automated iterative process, was encountered as a fast approach but with a severe drawback. Numerous stretches in the individual CDS sequences, where the read coverage was insufficient, were found crudely misassembled and needed manual cleaning. We, therefore, also restricted our use of the plastome to the protein-coding CDS and refrained from attempting to use the entire plastome, thus also including non-coding regions, for the phylogenetic analysis. This restriction must be seen as critical in the light of recently reported gene tree conflicts in phylogenies based on plastome data (e.g., Gonçalves et al., 2019; Walker et al., 2019; Cho et al., 2020; Köhler et al., 2020), which questions the assumption of the plastome as a single locus. Cho et al. (2020) recovered different topologies for deep branches in *Sonchus* comparing phylogenetic reconstruction based on the protein-coding CDS only, with one based on the entire plastome, and Köhler et al. (2020) even recorded conflicting topologies among major clades of Opuntioideae (Cactaceae), when exploring different assemblies of top-informative CDS markers. However, it cannot in all cases be excluded so far that non-biological causes are responsible for such conflicts. The non-coding portions of the plastome are characterized not only by a higher frequency of substitutions compared to the more conserved coding regions but also by microstructural mutations ranging from length-variable mononucleotide and other hypervariable stretches to inversions, providing a challenge for the correct assessment of the positional homology of the nucleotides. Employing correctly aligned non-coding regions, thus, becomes more mandatory and crucial, the lower the overall genetic distance (Escobari et al., 2021).

It seems at least a precautionary measure to extend the application of the multispecies coalescent approach also to the plastid DNA matrix. However, our reconstructions based on the protein-coding CDS show no hard topological incongruences that would indicate the presence of significantly discordant plastid gene trees, apart from generally lower support of the branches and a consequently lower resolution of the ASTRAL tree (Figures 4A,B, 7, Supplementary Figure 5, and Table 2). This result agrees with the general finding that in the Asteraceae the CDS regions are strongly conserved (e.g., Kim et al., 2005; Pascual-Díaz et al., 2021). Our exclusive use of the protein-coding CDS sequences limited the phylogenetic resolution, such as that shallow nodes, and, thus, relationships at the species level, frequently remained unresolved, apparently because relatively few genetic differences have accumulated among taxa of more recent origin.

### Hyb-Seq Provides Novel Insights Into the Evolutionary History of and Relationships Within Subtribe Scorzonerinae

Our phylogenetic reconstructions corroborate the monophyly of the Scorzonerinae and fully resolved its backbone phylogeny (Figure 3). Moreover, the taxon composition of the major lineages identified is highly consistent in all our nuclear and plastid DNA analyses (Figures 5–7); whereas the analyses of Sanger sequenced data by Zaika et al. (2020: Figures 1, 2) successfully identified phylogenetic lineages within Scorzonerinae, the relationships among lineages of the subtribe were mostly unresolved. In so far as our study corroborates the major lineages identified by Zaika et al. (2020) and resolved their relationships, it confirms the monophyly of the genera recognized by the authors in their revised generic taxonomy of the subtribe.

Our trees obtained from the nuclear datasets show robust support for the early divergence of two principal clades within the Scorzonerinae, one consisting of *Gelasia*, *Koelpinia*, *Pterachaenia*, and *Ramaliella* and the other of *Pseudopodospermum*, *Takhtajaninatha*, *Epilasia*, *Tragopogon*, *Geropogon*, and *Scorzonera* (Figure 3). The monospecific NW African *Tourneuxia*, for which sequences could not be generated, can be expected to be either sister to both clades, or sister to the members of the first clade, according to the findings by Zaika et al. (2020). Both principal clades are also present in NW Africa, which is estimated to belong to the ancestral area of the tribe, of which subtribe Scorzonerinae (Zaika et al., 2020) is one of the early diverging clades (Tremetsberger et al., 2012; Kilian et al., in prep.).

The Scorzonerinae have, however, developed their highest diversity in the E Mediterranean-SW to Middle Asian region. The further diversification of the first principal clade, with *Gelasia* and *Koelpinia* as successive sisters to a clade of *Pterachaenia* and *Ramaliella* (Figure 3), is centered in the E Mediterranean-SW to Middle Asian region, where the *Pterachaenia* and *Ramaliella* clades are exclusively found. The same holds for the second principal clade, where *Epilasia*, *Pseudopodospermum*, *Takhtajaniantha*, and *Scorzonera* are successive sisters to a *Tragopogon-Geropogon* clade (Figure 3). *Epilasia* and *Takhtajaniantha* are restricted to that area and the other lineages have their highest diversity there. The phylogeny of *Tragopogon* (Mavrodiev et al., 2004) clearly shows that the N African species evolved from later diversification and migration.

Tremetsberger et al. (2012) estimated the origin of the Scorzonerinae clade in the late Oligocene (c. 25 mya) and the diversification between *Gelasia* (represented in their study by *G. hirsuta*) and *Scorzonera* and *Tragopogon*, corresponding to the origin of the two principal clades, in the late Early Miocene (c. 17–18 mya). This age estimation for the diversification of the subtribe was corroborated by Fernández-Mazuecos et al. (2016) and coincides with the onset of a series of tectonic, orographic, and climatic changes in the Miocene. These led to an expansion of open vegetation and the formation of diversified mountain habitats and triggered diversification also in other subtribes (Tremetsberger et al., 2012; Kilian et al., 2017).

*Scorzonera* and *Tragopogon* are estimated by Tremetsberger et al. (2012) to have split in the transition between the Middle and Late Miocene (c. 11 mya). However, Bell et al. (2012), who analyzed diversification and diversification rates of *Tragopogon* with its >150 species, estimated the origin of that genus later in the Late Miocene c. 7.4 mya (3.7–11.6 HPD). The highest species diversity of *Tragopogon* is centered in the mountainous habitats around the Paratethys basin, where the Black, Caspian, and Aral Seas represent relics of the former Paratethys Sea. They estimated its diversification to have taken place rather late and rapidly from 2.6 mya onward, thus, well after the Messinian Salinity Crisis in the late Miocene between 5.3 and 5.96 mya, which brought about a dramatic aridization of the entire region (Bell et al., 2012). Regarding the distribution of *Tragopogon*, parallels can be drawn to some extent also for the other large genera of the subtribe (*Gelasia*, *Pseudopodospermum*, and *Scorzonera*). Furthermore, a rather recent diversification seems likely given the shallow genetic differences within these major lineages (see below).

The evolutionary history of the subtribe, however, must have been somewhat more complicated, considering the backbone tree based on the uniparentally inherited plastome CDS (Figures 4A,B). Lacking a sample of *Tourneuxia*, which was inferred as the first diverging clade of the subtribe in the plastid DNA tree by Zaika et al. (2020), our analyses resolved *Geropogon*, represented by two samples, as sister to the remainder of the subtribe (Figure 4). We assume that the *Tragopogon-Geropogon* clade is another early diverging clade of the subtribe in the plastome phylogeny because in both nuclear and plastid DNA phylogenies (Mavrodiev et al., 2012; Zaika et al., 2020) *Geropogon* is the well-supported sister group to *Tragopogon*, for which we could not assemble a plastome. The position of *Geropogon* in our plastid DNA tree is, therefore, in striking contrast to the topology of the nuclear phylogeny with the *Tragopogon-Geropogon* clade deeply nested in the principal clade with *Scorzonera* (Figure 3). We propose two potential explanations for this discrepancy: the plastome phylogeny may present the species phylogeny, encapsulating the early diverging origin of the *Geropogon-Tragopogon* lineage before it underwent reticulation with one lineage of the remainder of the subtribe. Alternatively, the nuclear phylogeny may present the species phylogeny and the *Geropogon-Tragopogon* lineage acquired the plastome from an early diverging lineage of the subtribe, e.g., *Gelasia*, by hybridization with extensive backcrossing, thus through chloroplast capture (Rieseberg and Soltis, 1991). The hybridization left no apparent morphological traces in the *Geropogon-Tragopogon* lineage due to the absence of significant nuclear gene flow. The clear morphological distinction of the *Geropogon-Tragopogon* lineage, compared to the remainder of the subtribe, which is evident by its uniseriate involucre, may provide an argument for the former scenario. The pollen type of *Tragopogon* shared with *Koelpinia*, and that of *Geropogon*, shared with a few members of *Gelasia* (*G. lanata* type; Blackmore, 1982, 1986) do not help to decide between the scenarios. The fact that *Lipschitzia*, the monotypic Central-E Asian genus accommodating the former *Scorzonera divaricata* and not represented in our analyses, is resolved, as sister to the *Tragopogon-Geropogon* clade in the plastid DNA tree by Zaika et al. (2020), may support the cytoplasmatic gene flow hypothesis. However, that *Lipschitzia* is not an early diverging lineage in the nuclear phylogeny by Zaika et al. (2020) further complicates the picture.

Within the large clade sister to *Geropogon* in the plastid DNA tree (Figures 4A,B), two main clades are resolved: one including *Takhtajaniantha* and *Scorzonera*, their sister group relationship is, however, not statistically supported in the coalescent analyses, while the other includes the remainder of Scorzonerinae. Zaika et al. (2020), found the same clades with mostly strong statistical support, and also the *Koelpinia-Pterachaenia-Ramaliella* and the *Epilasia-Pseudopodospermum* subclades in the second clade. In the nuclear trees, in contrast, *Scorzonera, Takhtajaniantha, Pseudopodospermum*, and *Epilasia* form consecutive sister groups to the *Geropogon-Tragopogon* clade (Figures 4A,B). The *Koelpinia-Pterachaenia-Ramaliella* clade is remarkable as the only major clade besides *Scorzonera*, the composition of which is congruent in both phylogenies.

Within the *Scorzonera* clade, an odd incongruence occurs with the monospecific *S. renzii* clade: in the plastid DNA tree it groups with the *S. purpurea-Podospermum* clade (ASTRAL tree) or the *S. purpurea-Podospermum-Scorzonera* s.str. clade (concatenation trees; also, in Zaika et al., 2020), but with the *S. rupicola-S. angustifolia-S. albicaulis* clade in the nuclear trees (Figures 4A,B). Therefore, the relationship of *S. renzii* remains an open question.

Different processes can cause cytonuclear discordance: ancestral polymorphism and population splitting resulting in incomplete lineage sorting, fixation of different organellar genomes from existing variation by the selection, or hybridization with cytoplasmic introgression (chloroplast capture; Lee-Yaw et al., 2019). Considering that the coalescent method rules out incomplete lineage sorting, our findings of massive nuclear gene tree conflicts and the high rate of paralogs may support the hypothesis that hybridization with cytoplasmic and/or nuclear introgression led to multiple events of reticulate evolution during both the early and later diversification within the Scorzonerinae. This is similar but distinctly more pronounced than what was concluded for the subtribe Lactucinae (Kilian et al., 2017).

The shallow morphological differentiation between the major Scorzonerinae lineages may also be of some significance in this context. As already noted by Zaika et al. (2020), morphology badly reflects the major lineages and genera, making their diagnoses burdensome. Most major lineages and clades lack non-homoplastic synapomorphies (Mavrodiev et al., 2004; Hatami et al., 2020; Zaika et al., 2020). Likewise, reliable diagnostic morphological characters seem largely absent for the two principal clades and their subclades. In contrast to the case of the Lactucinae, where similar sets of features evolved multiple times in different lineages (Kilian et al., 2017, in prep.), certain patterns of character state distribution across, more or less, distant clades in the Scorzonerinae may find a more parsimonious explanation through reticulation than convergent evolution. Pollen features (Blackmore, 1982, 1986) may illustrate this thought. The Scorzonerinae are unique among the tribe in this respect. Not only does the pollen possess colpori with only two lacunae in contrast to three in all other subtribes, but their pollen also exhibits seven distinctive morphological types. According to the hypothesis of character evolution provided by Blackmore (1982), they form four main developmental branches. The distribution of these pollen types in the nuclear backbone tree and their parsimony ancestral character reconstruction (Figure 4C) shows a conspicuous mixture of multiple state dimorphisms and dual or plural homoplasies in the terminals. Similar patterns could be elaborated for other morphological character sets and their comparisons could help to infer the parentage of hybrid lineages.

### Comparison of Interspecific Relationships Obtained From Different Datasets and Analyses

In principle, our study confirmed that the Hyb-Seq approach is useful to resolve close relationships at interspecific levels. However, branch support strongly decreased for internal nodes closer to the tips compared to backbone relationships. We interpret this because of the presumed young age of the speciation within the major lineages, which was inferred for *Tragopogon* by Bell et al. (2012) and can likely be generalized for the other larger lineages of the subtribe, *Scorzonera*, *Pseudopodospermum*, and *Gelasia*. In addition, we suspect that reticulation events between and within the major lineages have contributed to blurring interspecific relationships, as can be deduced from the comparatively low percentage of loci confirmed as orthologous across the subtribe (see above). Consequently, in many cases, infrageneric and interspecific relationships received no or low statistical support and showed incongruences among analyses, which, considering their lack of support, are not meaningful. Some species groups, in contrast, were consistently well-supported as monophyletic. In the following, we focus on these well-supported species groups, discussing their internal relationships, morphological synapomorphies, and possible occurrence of cytonuclear discordance.

***Gelasia:*** Both nuclear and plastid DNA analyses strongly support the ***Gelasia lanata* group** composed of *G. flaccida* (*S. flaccida* Rech. f.), *G. lanata* (L.) Zaika et al. (Figure 1D), and *G. psychrophila* (Boiss. & Hausskn.) Zaika et al. (BS ≥ 99, 1 PP, 1 LPP), although the relationships between these species remained unresolved (Figures 5–7 and Supplementary Figures 1–5). Morphologically, they possess a combination of the tuberous root, scape-like stems, and achenes with densely white lanate hairs. The ***Gelasia persica* group**, including *G. persica* (*S. persica* Boiss. & Buhse), *G. wendelboi* (*S. wendelboi* Rech. f.), and *G. xylobasis* (*S. xylobasis* Rech. f.) is also monophyletic in both nuclear and plastid analyses (BS ≥ 89, PP ≥ 0.95, LPP ≥ 0.9, Figures 5–7, and Supplementary Figures 1–5), except for the OLS and ELS coalescent and supercontig ML-unpartitioned-concatenation analyses. These three species are endemic to Iran and possess lanceolate or oblong leaves with three to five main veins and glabrous achenes. They share this combination of morphological characters with *G. tomentosa* (L.) Zaika et al. and *G. cinerea* (Boiss.) Zaika et al., which is consistent with the nuclear DNA analyses that placed the latter two species with the *G. persica* group in a moderately to the highly supported clade (BS ≥ 98, 1 PP, LPP ≥ 0.98, 73 BS-NP, 84 BS-P, Figures 5, 6 and Supplementary Figures 1–4). However, in the plastid DNA analyses, the *G. persica* group in its narrow sense formed instead a well-supported clade with *G. seidlitzii* (Boiss.) Zaika et al. (89 BS-NP, 95 BS-P, 0.95 PP, 1 LPP, Figure 7, and Supplementary Figure 5), thus, represent a clear case of cytonuclear discordance. A larger ***Gelasia villosa* group**, besides *G. villosa* (Scop.) Cass., which provides the type of the generic name, including *G. doriae* (Degen & Bald.) Zaika et al., *G. ensifolia* (M.Bieb.) Zaika et al., *G. biebersteinii* (Lipsch.) Zaika et al., *G. caespitosa* (Pomel) Zaika et al., and *G. callosa* (Moris) Zaika et al. were usually resolved with low to moderate support only but fairly consistently in both nuclear (67 BS-P, 55 BS-NP, 1 PP, 0.9 LPP, incompletely so in the supercontig analyses and ML-unpartitioned-concatenation analysis of OLS, Figures 5, 6 and Supplementary Figures 1–4) and plastid DNA analyses (88 BS-P, 59 BS-NP, 0.7 LPP, incompletely so in the Bayesian analysis, Figure 7, and Supplementary Figure 5). We have, however, not noticed morphological synapomorphies for this group.

***Pseudopodospermum:*** All nuclear and plastid DNA analyses resolved the ***Pseudopodospermum papposum* group**, including *P. ovatum* (Trautv.) Zaika et al., *P. ferganicum* (*S. ferganica* Krasch.), *P. papposum* (DC.) Zaika et al., *P. picridioides* (Boiss.) Hatami, and *P. limnophilum* (*S. limnophila* Boiss.), as monophyletic with high support (98 BS, 0.99 PP, 1 LPP, Figures 5–7, and Supplementary Figures 1–5). Monophyly of this group is also supported by morphology, as these species share a tuberous root, tuberculate achenes, and a pappus with entirely plumose bristles (Hatami and Mirtadzadini, 2022). Also, well-supported is the ***Pseudopodospermum undulatum* group** composed of *P. undulatum* (Vahl) Zaika et al., *P. brevicaule* (Vahl) Zaika et al., *P. baeticum* (DC.) Zaika et al., and *P. reverchonii* (Debeaux & Hervier) Zaika et al. (97 BS, 1 PP, 0.98 LPP, Figures 5, 6 and Supplementary Figures 1–4). Their internal relationships remain largely unresolved. The plastid DNA analyses resolved *P. reverchonii* as sister to *P. baeticum* (100 BS, 0.99 PP, 1 LPP, Figure 7, and Supplementary Figure 5) and *P. brevicaule* as sister to *P. undulatum* (100 BS, 1 PP, 0.98 LPP, Figure 7, and Supplementary Figure 5), but both clades are part of a larger polytomy, and their relationship is, thus, unresolved. The distribution of the species group is centered in the W Mediterranean, with *P. undulatum* extending through N Africa to the Arabian Peninsula, a geographic pattern that is exclusive to this species group in *Pseudopodospermum*. A third well-supported group in the nuclear DNA analyses is that of ***Pseudopodospermum incisum*** (DC.) Zaika et al., which also includes, apart from the name-giving species, *P. calyculatum* (Boiss.) Zaika et al., *P. bicolor* (*S. bicolor* Freyn & Sint.), and *P. violaceum* (D. F. Chamb.) Zaika et al. (BS ≥ 0.96, 1 PP, 1 LPP, Figures 5, 6 and Supplementary Figures 1–4); the plastid DNA analyses did not resolve this clade. The four species share dentate to pinnatisect leaves as exclusive synapomorphy in contrast to the entire leaves in all other species of *Pseudopodospermum*.

***Takhtajaniantha:*** All nuclear analyses strongly support a ***Takhtajaniantha austriaca* group** composed of *T. crispa* (M. Bieb.) Zaika et al., *T. grubovii* (*S. grubovii* Lipsch.) and *T. austriaca* (Willd.) Zaika et al. (100 BS, 1 PP, 1 LPP, Figures 5, 6 and Supplementary Figures 1–4), their internal relationships, however, were not well-supported. Also, well-supported in all nuclear analyses is the sister group relationship of *T. mongolica* (Maxim.) Zaika et al. and *T. pseudodivaricata* (Lipsch.) Zaika et al. (100 BS, 1 PP, 0.99 LPP, Figures 5, 6 and Supplementary Figures 1–4), having cauline stem leaves with the opposite arrangement as a synapomorphy. In all nuclear analyses, except the supercontig ones, the *T. austriaca* group clade is sister to a clade including the remainder of the *Takhtajaniantha* taxa of our analysis, thus, in addition to the latter two species, these are *T. capito* (Maxim.) Zaika et al., *T. ikonnikovii* (Krasch. & Lipsch.) Zaika et al., *T. pusilla* (Pall.) Nazarova, and *T. tau-saghyz* (Lipsch. & G. G. Bosse) Zaika et al.

***Scorzonera* s. typ. clade:** Among the four species of this clade, all nuclear (except the supercontig coalescent analysis) and plastid analyses support the sister group relationship of *S. radiata* Fisch. ex Ledeb. and *S. humilis* L. well (100 BS, 1 PP, LPP ≥ 0.99, Figures 5–7, and Supplementary Figures 1–5). Morphologically, this correlates with the red spot on the apex of phyllaries in both species. *Scorzonera aristata* stands also morphologically apart from tuberculate-ribbed achenes instead of the otherwise common smooth-ribbed achenes; *S. parviflora* is odd as a halophytic species with creeping rootstock.

***Scorzonera albicaulis* clade:** All nuclear analyses support a ***Scorzonera bracteosa* group** well, consisting of *S. bracteosa* C. Winkl., *S. crassicaulis* Rech. f., and *S. tragopogonoides* Regel & Schmalh. (BS ≥ 90, 1 PP, 1 LPP, Figures 5, 6 and Supplementary Figures 1–4). OLS Bayesian and ML-concatenation analyses of ELS also resolved the interspecific relationships of this group, with *S. bracteosa* as sister to *S. crassicaulis* and both as sister to *S. tragopogonoides*, while in the plastid analyses *S. tragopogonoides* was not included. The three species possess very large capitula (5–7 cm long at fruiting) and bracteal leaves below the capitula.

***Podospermum* clade:** In all nuclear and plastid analyses, the species relationships within the *Podospermum* clade are largely unresolved. The only congruent interspecific topology in all nuclear analyses and both plastid ML-concatenation analyses is the sister group relationship of *S. meshhedensis* (Rech. f.) Rech. f. and *S. songorica* (Kar. & Kir.) Lipsch. & Vassilcz. (100 BS, 1 PP, 1 LPP, Figures 5–7, and Supplementary Figures 1–5), already found in previous studies (Hatami et al., 2020; Zaika et al., 2020). Both species share leaf heteromorphy (entire and pinnatisect) and an easily detachable pappus.

All in all, the Hyb-Seq approach helped to clarify infrageneric and interspecific relationships in the Scorzonerinae but only in a few cases. Infrageneric classification of the major lineages, thus, remains a challenge, both as such and concerning a suitable methodological approach.

### Taxonomy

**New Combinations**

The following new combinations have become necessary for species confirmed by our analyses as members of phylogenetic lineages that are classified as separate genera of the Scorzonerinae following Zaika et al. (2020):

***Gelasia flaccida*** (Rech. f.) E. Hatami, N. Kilian & K.E. Jones, **comb. nov.** ≡ *Scorzonera flaccida* Rech. f., Fl. Iran. 122: 73. 1977.

***Gelasia persica*** (Boiss. & Buhse) E. Hatami, N. Kilian & K.E. Jones, **comb. nov.** ≡ *Scorzonera persica* Boiss. & Buhse in Mém. Soc. Imp. Naturalistes Moscou 12: 139. 1860.

***Gelasia subaphylla*** (Boiss.) E. Hatami, N. Kilian & K.E. Jones, **comb. nov.** ≡ *Scorzonera subaphylla* Boiss., Diagn. Pl. Orient., ser. 1, 7: 8. 1846.

***Gelasia veratrifolia*** (Fenzl) E. Hatami, N. Kilian & K.E. Jones, **comb. nov.** ≡ *Scorzonera veratrifolia* Fenzl in Flora 26: 399. 1843.

***Gelasia wendelboi*** (Rech. f.) E. Hatami, N. Kilian & K.E. Jones, **comb. nov.** ≡ *Scorzonera wendelboi* Rech. f., Fl. Iran. 122: 66. 1977.

***Gelasia xylobasis*** (Rech. f.) E. Hatami, N. Kilian & K.E. Jones, **comb. nov.** ≡ *Scorzonera xylobasis* Rech. f., Fl. Iran. 122: 66. 1977.

***Pseudopodospermum bicolor*** (Freyn & Sint.) E. Hatami, N. Kilian & K.E. Jones, **comb. nov.** ≡ *Scorzonera bicolor* Freyn & Sint. in Österr. Bot. Z. 43: 266. 1892.

***Pseudopodospermum ferganicum*** (Krasch.) E. Hatami, N. Kilian & K.E. Jones, **comb. nov.** ≡ *Scorzonera ferganica* Krasch. in Trudy Bot. Inst. Akad. Nauk SSSR, Ser. 1, Fl. Sist. Vysš. Rast. 1: 180. 1933.

***Pseudopodospermum lacerum*** (Boiss. & Balansa) E. Hatami, N. Kilian & K.E. Jones, **comb. nov.** ≡ *Scorzonera lacera* Boiss. & Balansa in Boissier, Diagn. Pl. Orient., ser. 2, 5: 116. 1856.

***Pseudopodospermum limnophilum*** (Boiss.) E. Hatami, N. Kilian & K.E. Jones, **comb. nov.** ≡ *Scorzonera limnophila* Boiss., Diagn. Pl. Orient., ser. 1, 7: 7. 1846.

***Pseudopodospermum nivale*** (Boiss. & Hausskn.) E. Hatami, N. Kilian & K.E. Jones, **comb. nov.** ≡ *Scorzonera nivalis* Boiss. & Hauskn. in Boissier, Fl. Orient. 3: 765. 1875.

***Pseudopodospermum tunicatum*** (Rech. f. & Köie) E. Hatami, N. Kilian & K.E. Jones, **comb. nov.** ≡ *Scorzonera tunicata* Rech. f. & Köie in Biol. Skr. 8,2: 196. 1955.

***Ramaliella microcalathia*** (Rech. f.) E. Hatami, N. Kilian & K.E. Jones, **comb. nov.** ≡ *Scorzonera tortuosissima* var. *microcalathia* Rech. f. in Ann. Naturhist. Mus. Wien 55: 291. 1944 ≡ *Scorzonera microcalathia* (Rech. f.) Rech. f. in Anz. Österr. Akad. Wiss., Math.-Naturwiss. Kl. 98: 248. 1961.

***Takhtajaniantha grubovii*** (Lipsch.) E. Hatami, N. Kilian & K.E. Jones, **comb. nov.** ≡ *Scorzonera grubovii* Lipsch. in Novosti Sist. Vyssh. Rast. 18: 229. 1981.

**Revised infrageneric classification of Scorzonera**

Maintaining the circumscription of *Scorzonera* by Zaika et al. (2020), which is confirmed by our reconstruction, we propose the following revised infrageneric classification.

**1. *Scorzonera* sect. *Rupicolae*** E. Hatami, N. Kilian & K.E. Jones, **sect. nov.** – Type: *S. rupicola* Hausskn.

**Diagnosis.** Suffruticose, dense pulvinate-caespitose perennial; stem with few or reduced leaves; capitula few on a single stem, easily falling; achenes attenuated at the apex.

**Note.** *S. rupicola* was resolved already by Zaika et al. (2020) in a clade of its own. This suffruticose perennial has reduced leaves, easily falling capitula, and beaked achenes (Figure 1F). The combination of these morphological characters is unique in *Scorzonera*. Moreover, *S. rupicola* has no close allies in the genus.

**2. *Scorzonera* sect. *Renzianae*** E. Hatami, N. Kilian & K.E. Jones, **sect. nov.** – Type: *S. renzii* Rech. f.

**Diagnosis.** Uppermost leaves filiform, spreading, subtending the capitula; capitula with short peduncles or subsessile in a racemiform synflorescence; phyllaries thick membraneous, subkeeled by ± prominent midrib.

**Note.** *S. renzii* was resolved as the only species of the “*S. renzii* clade” in Zaika et al. (2020) and our analyses. Morphologically, the combination of racemiform synflorescence, capitula with subtending leaves, and thick membraneous phyllaries with ± prominent subkeeled midrib are exclusive to this species in *Scorzonera*. Additionally, our analyses show this species to have no closer allies.

**3. *Scorzonera* sect. *Piptopogon*** C. A. Mey. ex Turcz. in Bull. Soc. Imp. Naturalistes Moscou 21(3): 97. 1848 ≡ *Scorzonera* subg. *Piptopogon* (C. A. Mey. ex Turcz.) C. Díaz & Blanca in Anales Jard. Bot. Madrid 43: 330. 1987. – Type: *Scorzonera macrosperma* Turcz. ex DC. [= *S. albicaulis*]

= *Achyroseris* Sch. Bip. in Nov. Actorum Acad. Caes. Leop.-Carol. Nat. Cur. 21: 165. 1845. – Type: *Achyroseris macrosperma* (Turcz. ex DC.) Sch.Bip. [=*S. albicaulis* Bunge]
= *Scorzonera* sect. *Macrospermae* Nakai in Rep. Inst. Sci. Res. Manchoukuo, ser. 1, 6: 168. 1937 ≡ *Scorzonera* ser. *Macrospermae* Lipsch. in Bobrov & Tzvelev, Fl. URSS 29: 719. 1964. – Type: *Scorzonera albicaulis* Bunge
= *Scorzonera* ser. *Acanthocladae* Lipsch. in Bobrov & Tzvelev, Fl. URSS 29: 722. 1964. – Type: *Scorzonera acanthoclada* Franch.
= *Scorzonera* ser. *Bracteosae* Lipsch. in Bobrov & Tzvelev, Fl. URSS 29: 720. 1964. – Type: *Scorzonera bracteosa* C. Winkl.
= *Scorzonera* ser. *Franchetianae* Lipsch. in Bobrov & Tzvelev, Fl. URSS 29: 721. 1964. – Type: *Scorzonera franchetii* Lipsch.
= *Scorzonera* ser. *Pauciflorae* Lipsch. in Bobrov & Tzvelev, Fl. URSS 29: 721. 1964. – Type: *Scorzonera turkestanica* Franch.
= *Scorzonera* ser. *Tragopogonoideae* Lipsch. in Bobrov & Tzvelev, Fl. URSS 29: 720. 1964. – Type: *Scorzonera tragopogonoides* Regel & Schmalh.
= *Scorzonera* sect. *Turkestanicae* Lipsch. in Bobrov & Tzvelev, Fl. URSS 29: 720. 1964. – Type: *Scorzonera turkestanica* Franch.

**Diagnosis.** Perennial herbs or subshrubs; leaves graminoid; capitula with 4–12 flowers only; achenes without carpopodium, beaked or at least attenuate at apex; pappus dirty yellow, caducous; pollen with 24 lacunae.

**Note.** All nuclear and plastid analyses support the sister relationship of the *S. angustifolia* and *S. albicaulis* clades and the members of these clades also share morphological similarities, particularly having the same pollen type and apically attenuate to beaked achenes. The section has been recognized for a long time. It was also resolved as a separate clade by Zaika et al. (2020) with some 13 species. It is here confirmed to also contain *S. angustifolia* L.

**4. *Scorzonera* sect. *Scorzonera***

= *Scorzonera* sect. *Parviflorae* Lipsch. in Bobrov & Tzvelev, Fl. URSS 29: 79. 1964 ≡ *Scorzonera* ser. *Parviflorae* Lipsch. in Bobrov & Tzvelev, Fl. URSS 29: 720. 1964. – Type: *Scorzonera parviflora* Jacq.

= *Scorzonera* sect. *Radiatae* Nakai in Rep. Inst. Sci. Res. Manchoukuo, ser. 1, 6: 169. 1937. – Type: *Scorzonera radiata* Fisch. ex Ledeb.

**Diagnosis.** Leaves entire; capitula solitary or by a few only; inner phyllaries with apical red or blackish spots; achenes without carpopodium.

**Note.** The typical section includes only four species: *Scorzonera aristata*, *S. humilis*, *S. parviflora*, and *S. radiata* (inclusion assumed by Zaika et al. (2020) and confirmed by our study).

**5. *Scorzonera* sect. *Podospermum*** (DC.) Benth. in Bentham & Hooker, Gen. Pl. 2: 532. 1873 ≡ *Podospermum* DC. in Lamarck & Candolle, Fl. Franç., ed. 3, 4: 61. 1805, nom. cons. ≡ *Scorzonera* subg. *Podospermum* (DC.) Lipsch., Fragm. Monogr. Gen. Scorzonera 1: 7. 1935. – Type: *Scorzonera laciniata* L.

= *Scorzonera* sect. *Purpurea* Lipsch., Fragm. Monogr. Gen. Scorzonera 2: 104. 1939. – Type: *Scorzonera purpurea* L.

**Diagnosis.** Leaves (at least some) pinnately divided, more rarely graminoid; phyllaries subapically often corniculate; achene with conspicuous tubular carpopodium; achene surface mostly glabrous or somewhat hairy.

**Note.** The sister-group relationship of the *S. purpurea* and *Podospermum* clades was resolved with high support in all nuclear analyses and received at least weak to moderate support also in the plastid DNA analyses, and the presence of a tubular carpopodium in the achenes is a synapomorphy for the *Podospermum-S. purpurea* clade. We take this as justification for treating them in a single section. Within this section, the members of the *S. purpurea* clade are, nevertheless, set apart by their caudex with (instead of lacking) blackish-brown fibrous leaf sheath residues, graminoid leaves (instead of having at least some pinnately divided leaves), and pink or purplish (instead of yellow) flowers. These morphological differences may, however, be taken as justification to treat both clades alternatively as separate sections.

## Data Availability Statement

The datasets presented in this study can be found in online repositories. The names of the repositories and accession numbers are given in the Appendix.

## Author Contributions

NK and KJ designed the project. KJ and EH performed the laboratory work and generated data. NK and EH conducted post-sequencing data processing and phylogenetic analyses, discussed the results, and wrote the manuscript. KJ commented on and revised the manuscript. All authors read and approved the final manuscript.

## Conflict of Interest

The authors declare that the research was conducted in the absence of any commercial or financial relationships that could be construed as a potential conflict of interest.

## Publisher’s Note

All claims expressed in this article are solely those of the authors and do not necessarily represent those of their affiliated organizations, or those of the publisher, the editors and the reviewers. Any product that may be evaluated in this article, or claim that may be made by its manufacturer, is not guaranteed or endorsed by the publisher.

## Appendix

### Appendix | Sample data

Data for samples of which the sequences were newly generated in this study are provided in the following sequence: taxon name, B:DNA Bank numbers used in the phylogenetic trees, INSDC (International Nucleotide Sequence Database Collaboration) accession number for the deposited sequence data, locality, collector(s), collecting date, specimen barcode with stable specimen URI, if available, or herbarium code only). Samples with an INSDC accession number only represent sequence data that were already published.

**Outgroup**

*Catananche arenaria* Coss. and Durieu, DB473, ERX9058743, Tunisia, Gouvernorat de Gafsa, 9 May 1994, R. Vogt and C. Oberprieler, B 10 0209169. — *Chondrilla ramosissima* Sm., DB303, ERX9058744, Greece, Korinthia, 1 Oct 2003, R. Willing and E. Willing, B 10 0142299. — *Cichorium intybus* L., DB33105, ERX9058745, Greece, Dig House, 18 Jun 1971, J. M. Shay, B 10 9011338; MK569377. — *Hypochaeris achyrophorus* L., DB546, ERX9058774, Cyprus, Kritou Tera, 1 May 2007, R. Hand, B 10 0209668. — *Lactuca sativa* L., DQ383816; NC007578. —*Leontodon tingitanus* (Boiss. and Reut.) Ball, SRX5893068. — *Scolymus hispanicus* L., SRX5893152. — *Urospermum dalechampii* (L.) F. W. Schmidt, DB33108, ERX9058871, Italy, Sizilien, Caltanisetta, 6 Jun 1988, M. Erben, B 10 0677926. — *Willemetia stipitata* (Jacq.) Dalla Torre, DB33114, ERX9058872, Austria, Flora des Dachsteingebietes, 17 Jul 1955, F. Morton, B 10 0110511.

**Scorzonerinae**

*Epilasia hemilasia* (Bunge) Kuntze, DB32184, ERX9058716, Uzbekistan, Taschkent (wild source), cult. in Royal Botanical Garden Edinburgh, 17 May 1973, coll. anon., B 10 0541096. — *E. mirabilis* Lipsch., DB6491, ERX9058746, Afghanistan, Kataghan, 5 May 1967, K. H. Rechinger, B 10 0355010. — *Gelasia biebersteinii* (Lipsch.) Zaika et al., DB32212, ERX9058747, Azerbaijan, distr. Nucha, 22 May 1936, A. Grossheim, B 10 0626125. — *G. caespitosa* (Pomel) Zaika et al., DB33118, ERX9058748, Morocco, Middle Atlas, Timahdite, 12 Jun 1992, B. Valdés et al., B 10 0348202. — *G. callosa* (Moris) Zaika et al., DB33147, ERX9058749, Italy, Sizilien, Prov. Palermo, 27 Apr 1994, M. Erben, B 10 0663907. — *G. cinerea* (Boiss.) Zaika et al., DB 44318, ERX9058750, Iran, Azarbaijan, 15 km S of Tshaldoran, 1 km N of Qaratshi-Bolaghi village, 19 Aug 2001, M. Mirtadzadini, B 10 1115544 + MIR 2252. — *G. doriae* (Degen and Bald.) Zaika et al., DB32195, ERX9058751, Greece, Etolia-Akarnania, 16 May 1991, R. Willing and E. Willing, B 10 1015411. — *G. ensifolia* (M.Bieb.) Zaika et al., DB6450, ERX9058752, Russia, Sarepta, 1896, A. Becker, B 10 0326679. — *G. flaccida* (Rech. f.) E. Hatami et al., DB 44327, ERX9058753, Iran, Bakhtiari, SW of Naghan, 30 May 2017, M. Mirtadzadini, B 10 1115549 + MIR 2317. — *G. lanata* (L.) Zaika et al., DB 44326, ERX9058754, Iran, Fars, Fasa, 26 Apr 2017, E. Hatami, B 10 1115550 + MIR 2996. — *G. latifolia* (Fenzl) E. Hatami et al., DB 44319, ERX9058755, Iran, 25 km from Zanjan to Abbar, 7 Jul 2016, M. Mirtazadini et al., MIR 2253; DB 44331, ERX9058756, Iran, West, Kermanshah, Quriqala, Mt. Shahu, 23 Aug 2007, M. Mirtadzadini, MIR 2258.—*G. persica* (Boiss. and Buhse) E. Hatami et al., DB 44323, ERX9058757, Iran, Central Alborz, Between Gatshar and Siahbisheh, southern slope of Kandavan pass, 21 Jul 2010, M. Mirtadzadini, B 10 1115513 + MIR 2257. — *G. psychrophila* (Boiss. and Hausskn.) Zaika et al., DB 44332, ERX9058758, Iran, West, Bakhtiari, 30 May 2017, M. Mirtadzadini, B 10 1115548 + MIR 2319. — *G. pygmaea* (Sm.) Zaika et al., DB32221, ERX9058759, Turkey, Isparta, Konya, 15 Jul 1999, M. Döring et al., B 10 0204866; DB32222, ERX9058760, Turkey, Nigde, 27 July 1999, M. Döring et al., B 10 0089921. — *G. ramosissima* (DC.) Zaika et al., DB 44350, ERX9058761, Iran, Kerman, NE of Shahr-e Babak, NE of Koron valley, on rocky slopes, 15 May 2014, M. Mirtazadini, B 10 1115551 + MIR 2309. — *G. rigida* (Aucher ex DC.) Zaika et al., DB 44259, ERX9058762, Iran, Azarbaijan, West of Khoi before Qotur, Taryamish village, on the tops of mountains, 5 July 2013, M. Mirtazadini, MIR 2226. — *G. seidlitzii* (Boiss.) Zaika et al., DB 44321, ERX9058763, Iran, Azarbaijan, NE of Khalkhal, Agh-Bolagh village, N slopes of Ajam, 9 Aug 2012, M. Mirtazadini, B 10 1115515 + MIR 2296.—*G. sericea* (DC.) Zaika et al., DB6441, ERX9058764, Turkey, Adana, Aladaglari, 7 Aug 1999, M. Döring et al., B 10 0204867.—*G. subaphylla* (Boiss.) E. Hatami et al., DB 44316, ERX9058765, Iran, Isfahan, on the pass 6 km NW of Semirom to Vanak, 19 Jun 2014, M. Mirtazadini, B 10 1115518 + MIR 2255. — *G. tuberosa* (Pall.) Zaika et al., DB33121, ERX9058767, Russia, Saratow, Sarepta, 1 Jan 1899, A. Becker, B 10 1015476. — *G. veratrifolia* (Fenzl) E. Hatami et al., DB6473, ERX9058768, Iraq, Kurdistan, Mosul, 9 Jul 1957, K. H. Rechinger, B 10 0326694. — *G. tomentosa* (L.) Zaika et al., DB32223, ERX9058766, Turkey, B6 Sivas, 4 Aug 1997, P. Hein, B 10 0664378. — *G. villosa* (Scop.) Cass., DB32226, ERX9058769, Slowenia, Istrien, 29 May 2005, W. Starmühler, B 10 0455872. — *G. wendelboi* (Rech. f.) E. Hatami et al., DB 44377, ERX9058770, Iran, Mazandaran, Nezva kuh area, between Orim and Taru, 2100 m, 6 Jul 1959, P. E. B. Wendelbo, W1965-0015341. —*G. xylobasis* (Rech. f.) E. Hatami et al., DB 44375, ERX9058771, Iran, Semnan, Shahmirzads, chashm, Kuhe Nizva (2800m), 1 Aug 1972, M. Iranshar and Zargani, W1973-0003571.—*Geropogon hybridus* (L.) Sch. Bip., DB33131, ERX9058772, Tunisia, Gouvernorat de Jendouba, 20 May 1994, R. Vogt and C. Oberprieler, B 10 0273204; DB33132, ERX9058773, Iran, 60 km NO versus Dezful, 19 19 Apr 1937, M. Køie, B 10 1015469. — *Koelpinia linearis* Pall., DB32188, ERX9058775, Turkey, Ankara, 5 May 2006, E. Bergmeier et al., B 10 0418791.—*K. tenuissima* Pavlov and Lipsch., DB33149, ERX9058776, Iran, Khorassan, 8 May 1975, K. H. Rechinger, B 10 1015463.—*Pseudopodospermum baeticum* (DC.) Zaika et al., DB32180, ERX9058779, Spain, prov. Malaga, 12 Mar 1979, P. Canto et al., B 10 1015405. — *P. bicolor* (Freyn and Sint.) E. Hatami et al., DB 44376, ERX9058778, Armenia, Vayots Dzor province, 16 Jun 2009, K. Kugler and E. Vitek, W2010-0003353. — *P. brevicaule* (Vahl) Zaika et al., DB32235, ERX9058780, Algerie, dep. Bouira, 22 Jun 1984, A. Dubuis, B 10 1015435. — *P.*aff.*calyculatum* (Boiss.) Zaika et al., DB 44300, ERX9058777, Iran, Fars, 17 km to Estahban from Eij, 25 Apr 2017, F. Bordbar, B 10 1115525 + MIR 2209. — *P. calyculatum* (Boiss.) Zaika et al., DB 44301, ERX9058781, Iran, Tehran, Firouz Kuh, after Lozour, 19 km from Arjman to Kariz, 30 Jul 2012, M. Mirtadzadini, B 10 1115556 + MIR 2205; DB 44306, ERX9058782, Iran, Gilan, 4 km from Jirandeh to Damash, 20 May 2016, M. Mirtadzadini, B 10 1115511 + MIR 2208; DB 44307, ERX9058783, Iran, Kermanshah, 26 km Hersin to Nurabad, 1 km after Ja’farabad, 23 May 2013, M. Mirtadzadini, MIR 2232; DB 44349, ERX9058784, Iran, Lorestan, Aleshtar, Dareh-Tang, 31 May 2016, M. Mirtadzadini, MIR 2246. — *P. davisii* (Lipsch.) Zaika et al., DB32216, ERX9058785, Iraq, Distr. Mosul (Kurdistan), 2 Jul 1957, K. H. Rechinger, B 10 0673664. — *P. elatum* (Boiss.) Zaika et al., DB6452, ERX9058786, Turkey, Antalya, 1 May 2002, Ö. Eren, B 10 0113389.—*P. ferganicum* (Krasch.) E. Hatami et al., DB33119, ERX9058787, Afghanistan, Gardez, 3 Jun 1967, K. H. Rechinger, B 10 1015478. — *P. hispanicum* (L.) Zaika et al., DB11495, ERX9058788, Germany, Thüringen, 18 Jul 2014, R. Hand and E. von Raab-Straube, B 10 0553282. — *P. hissaricum* (C. Winkl.) Zaika et al., DB33155, ERX9058789, Tajikistan, Pamir, Hissarksi khrebet, 40km situ septentrionali ab oppido Dushanbe, 23 May 1974, V. Vašák, B 10 1015457. —*P. idaeum* (Gand.) Zaika et al., DB33162, ERX9058790, Greece, Kreta, Dhikti-Gebirge, 19 May 1985, B. Egli, B 10 0703054. — *P. incisum* (DC.) Zaika et al., DB 44303, ERX9058791, Iran, Fars, between Estahban and Fasa, Khir valley, 4 May 2016, M. Mirtadzadini et al., B 10 1115520 + MIR 2221; DB 44363, ERX9058792, Iran, W, Bakhtiari, Swof Boldaji town, E of Owregan vill., 29 May 2017, M. Mirtadzadini, B 10 1115521 + MIR 2237. — *P. lacerum* (Boiss. and Balansa) E. Hatami et al., DB 44383, ERX9058793, Turkey, Adana, 5 June 1973, F. Sorger, W1992-0010067. — *P. limnophilum* (Boiss.) E. Hatami et al., DB6488, ERX9058794, Afghanistan, Ghazni, 18 Jul 1967, K. H. Rechinger, B 10 0355007. — *P. molle* (M. Bieb.) Kuth., DB33161, ERX9058796, Turkey, Mugla, Sandras Dagi above Agla, 22 Jun 1999, M. Döring et al., B 10 0204875; DB32233, ERX9058795, Greece, Kastoria, 6 May 2004, R. Willing and E. Willing, B 10 0178302. —*P. mucidum* (Rech. f. et al.) Zaika et al., DB 44311, ERX9058799, Iran, Fars, Darab, Gardaneye, Shekar-Morvarid, 2 May 2018, M. Mirtadzadini, B 10 1115555 + MIR 2292; DB 44294, ERX9058798, Iran, Fars, Darab, Gardaneye Shekar Morvarid, 2 May 2018, M. Mirtadzadini, B 10 1115554 + MIR 2260; DB 44267, ERX9058797, Iran, Kerman, Ravar, Hamkar mine, valley of Kor river, 19 Apr 2004, M. Mirtadzadini, B 10 1115516 + MIR 2194. — *P. nivale* (Boiss. and Hausskn.) E. Hatami et al., DB 44373, ERX9058800, Iran, Kurdistan, in montibus calcareis Avroman et Shahu., 1 Jun 1867, H. K. Haussknecht, W0051304. — *P. ovatum* (Trautv.) Zaika et al., DB 44351, ERX9058801, Iran, Baluchistan, NE of Bazman, Siah Band mt. range, 28 Apr 2017, M. Mirtadzadini, B 10 1115572 + MIR 2322. — *P. pachycephalum* (Podlech and Rech. f.) Zaika et al., DB33117, ERX9058802, Pakistan, Quetta, 8 May 1965, K. H. Rechinger, B 10 1015479. — *P. papposum* (DC.) Zaika et al., DB7158, ERX9058803, Israel, Shefela, 24 Mar 2010, M. Ristow, B 10 0355381. — *P. picridioides* (Boiss.) Hatami, DB 44329, ERX9058805, Iran, Fars, 25 km to Niriz from Khir, 25 Apr 2017, F. Bordbar, B 10 1115524 + MIR 2323.—*P. phaeopappum* (Boiss.) Zaika et al., DB 44286, ERX9058804, Iran, Kordestan, S of Marivan between Daraki and Tata pass, 24 May 2016, M. Mirtadzadini et al., B 10 1115560 + MIR 2162. — *P. raddeanum* (C. Winkl.) Zaika et al., DB 44281, ERX9058809, Iran, Kerman, Kheyr Abad, 1 Apr 2005, M. Mirtadzadini, B 10 1115559 + MIR 2191a; DB 44260, ERX9058807, Iran, Fars, the pass between Estahban and Niriz (old road), 3 May 2016, M. Mirtadzadini et al., B 10 1115570 + MIR 2157; DB 44293, ERX9058810, Iran, Kerman, SE of Sardu, E of Sarbizan pass, 29 Apr 2016, M. Mirtadzadini, MIR 2163; DB 44276, ERX9058808, Iran, Khorassan, Bojnurd, Gharlagh, 12 May 1992, Faghihnia and Zangooei, FUMH 21740; DB 44299, ERX9058811, Iran, Fars, 10 km to Niriz from Estahban, 25 Apr 2017, F. Bordbar, MIR 2177; DB32229, ERX9058806, Pakistan, Quetta, 11 May 1965, K. H. Rechinger, B 10 1015431. —*P. reverchonii* (Debeaux and Hervier) Zaika et al., DB32220, ERX9058812, Spain, province Jaen, Sierra de Cazorla, 1 May 1901, E. Reverchon, B 10 1015427. — *P. semicanum* (DC.) Zaika et al., DB 44378, ERX9058813, Turkey, B9 Aðri: 2 km SW of Hemur (Nurat valley), 1 Jun 1966, P. H. Davis, W 1976-0007423. — *P. szowitzii* (DC.) Kuth., DB 44263, ERX9058814, Iran, N of Tehran, on the hill at north of Dizin pass, 1 Jun 2013, A. Ebrahimi 2994, B 10 1115537 + MIR 2159; DB 44338, ERX9058816, Iran, East Azarbaijan province, Tabriz to Mianeh,, 5 May 2016, A. Ebrahimi, B 10 1115522 + MIR 2336; DB 44277, ERX9058815, Iran, E of Tehran, Haraz road, on stony hills beside Imam Zade-Haashem’s Tomb, 12 June 2015, M. Mirtadzadini, MIR 2169; DB 44346, ERX9058817, Iran, East Azarbaijan province, Tabriz to Shabestar, route of Sarkandizaj village, Mishodaghi, 12 May 2016, A. Ebrahimi, MIR 2346. —*P. tunicatum* (Rech. f. and Köie) E. Hatami et al., DB6474, ERX9058818, Afghanistan, Panjao, 26 Jun 1967, K. H. Rechinger, B 10 0326695. — *P. turkeviczii* (Krasch. and Lipsch.) Kuth., DB 44336, ERX9058821, Iran, East Azarbaijan province, Tabriz to Marand, Mishodaghi, 22 Apr 2016, A. Ebrahimi, B 10 1115562 + MIR 2339; DB 44334, ERX9058820, Iran, East Azarbaijan province, Tabriz to Espiran road, Kordkandi village, 6 May 2016, A. Ebrahimi, MIR 2332; DB 44291, ERX9058819, Iran, N of Fars province, the pass between Eqlid and Khonjesht, 27 Apr 2007, M. Mirtadzadini, B 10 1115552 + MIR 2164. — *P. undulatum* (Vahl) Zaika et al., DB32228, ERX9058822, Morocco, Middle Atlas, 28 Apr 1993, R. Vogt and C. Oberprieler, B 10 1015430. — *P. violaceum* (D. F. Chamb.) Zaika et al., DB6472, ERX9058823, Turkey, Konya, Ermenek to Hadim, 10 Jul 2000, Ö. Eren and G. Parolly, B 10 0204872. — *Pterachaenia stewartii* (Hook. f.) R. R. Stewart, DB33127, ERX9058824, Pakistan, Quetta, 20 May 1965, K. H. Rechinger, B 10 0001070. — *P. codringtonii* (Rech. f.) Zaika et al., DB 44113, ERX9058717, Afghanistan, Ghazni, 20 Jul 1967, K. H. Rechinger, B 10 1013632.—*Ramaliella intricata* (Boiss.) Zaika et al., DB 44308, ERX9058826, Iran, Kerman, on the way of Sarchesmeh to Rafsanjan, s.d., M. Kaleghi Yekta, B 10 1115545 + MIR 2399. — *R. longipapposa* (Rech. f.) Zaika et al., DB32214, ERX9058827, Iran, Baluchistan, 2 May 1977, K. H. Rechinger, B 10 1015425. — *R. microcalathia* (Rech. f.) E. Hatami et al., DB33160, ERX9058828, Iran, Khorassan, 29 May 1977, J. Renz and H. Runemark, B 10 1015454. — *R. musilii* (Velen.) Zaika et al., DB32193, ERX9058718, Yemen, environment of the town Rada, 12 Apr 1997, N. Kilian et al., B 10 0220796. — *R. polyclada* (Rech. f. and Köie) Zaika et al., DB32191, ERX9058719, Afghanistan, Kabul, 21 Jun 1965, K. H. Rechinger, B 10 1015409. —*R. tortuosissima* (Boiss.) Zaika et al., DB 44309, ERX9058829, Iran, Kerman, NE of Jiroft, between Saghder and Jiroft, Zarin village, 9 May 2013, M. Mirtadzadini, B 10 1115517 + MIR 2254. — *R.*aff.*tortuosissima* (Boiss.) Zaika et al., DB 44310, ERX9058825, Iran, Fars, NW of Bovanat, Jowlani village, 24 July 2011, M. Mirtadzadini, B 10 1115519 + MIR 2266. — *Scorzonera acanthoclada* Franch., DB33123, ERX9058830, Uzbekistan, Samarkand, Alpes Sarawschan, 21 Jul 1913, J. Bornmüller, B 10 1015474.—*S. alpigena* (K. Koch) Grossh., DB33133, ERX9058831, Cyprus, Larnaca, s.d., G. Alziar et al., B 10 1015468. — *S. angustifolia* L., DB32179, ERX9058832, Spain, SE, 1104/88, 20 Jun 1988, B. Valdes et al., B 10 1015404.—*S. aristata* DC., DB32185, ERX9058720, Spain, prov. Huesca, 14 Apr 1991, D. Gómez, B 10 0356367. — *S. armeniaca* (Boiss. and A. Huet) Boiss., DB 44342, ERX9058833, Iran, East Azarbaijan province, Tabriz to Espiran road, after Espiran, 29 Apr 2016, A. Ebrahimi, MIR 2342; DB 44365, ERX9058834, Iran, East Azarbaijan province, old road of Tabriz to Khajeh, Pakchin village, 29 Apr 2016, A. Ebrahimi 2995, B 10 1115547 + MIR 2295. — *S. bracteosa* C. Winkl., DB33158, ERX9058835, Uzbekistan, Samarkand,, 13 Jul 1913, J. Bornmüller, B 10 1015455.—*S. cana* (C. A. Mey.) Griseb., DB33142, ERX9058838, Greece, Evvia, Gerondas, 2 May 2011, R. Willing and E. Willing, B 10 0407427; DB33141, ERX9058837, Greece, Nom. Kavala, 26 Jun 1992, R. Willing and E. Willing, B 10 1015465; DB 44355, ERX9058840, Iran, East Azarbaijan province, Tabriz to Espiran road, Kordkandi village, 6 May 2016, A. Ebrahimi, MIR 2341; DB33139, ERX9058836, Austria, [> 1918], Woloszczak, B 10 1015466; DB 44315, ERX9058839, Iran, Khorassan, SW of Bojnurd, Raein, on Marjan rangeland road, Bali, 28 May 2006, Memariani and Zangooie, FUMH 37809. — *S. crassicaulis* Rech. f., DB33156, ERX9058841, Afghanistan, Bamian, 24 Jul 1962, K. H. Rechinger, B 10 1015456. — *S. graminifolia* L., DB32190, ERX9058721, Spain, Mtilla de los Cafios del Rio, 29 Jun 1984, S. Sánchez et al., B 10 1015408. — *S. grossheimii* Lipsch. and Vassilcz., DB32236, ERX9058842, Iran, Gorgan, Almeh, 8 Jun 1975, K. H. Rechinger, B 10 1015436. — *S. humilis* L., DB33159, ERX9058844, Germany, Franken, Windigholz bei Megesheim, 31 May 1960, H. Scholz, B 10 0304245; DB10497, ERX9058843, Germany, Schl. Holst., Kr. Eckernförde, 30 May 1969, G. Frahm, B 10 0349936. — *S. kandavanica* Rech. f., DB 44359, ERX9058845, Iran, Gilan, 5 km from Jirandeh to Damash, 20 May 2016, M. Mirtadzadini, B 10 1115542 + MIR 2214.—*S. laciniata* L., DB 44268, ERX9058847, Iran, Zanjan to Bijar, 11 km from Zarinabad to Halab, 22 May 2016, M. Mirtadzadini, B 10 1115569 + MIR 2216; DB33163, ERX9058846, Greece, Nom. Magnesia, 10 May 1993, R. Willing and E. Willing, B 10 1015453.—*S. luristanica* Rech. f., DB 44272, ERX9058848, Iran, Kermanshah, between Quriqala and Paweh, 26 May 2016, M. Mirtadzadini et al., B 10 1115571 + MIR 2213; DB 44313, ERX9058849, Iran, Lorestan, Aleshtar, NW of Firuzabad, mt. Khiat, 4 June 2017, M. Mirtadzadini, B 10 1115553 + MIR 2238. — *S. meshhedensis* (Rech. f.) Rech. f., DB 44257, ERX9058850, Iran, Kerman to Bam road, Golbaf, near Abolfazl Mosque, 14 Apr 2016, M. Mirtadzadini, B 10 1115557 + MIR 2244. — *S. meyeri* (K. Koch) Lipsch., DB 44335, ERX9058851, Iran, East Azarbaijan province, old road of Tabriz to Mianeh, 15 km before to Mianeh, 22 Apr 2016, A. Ebrahimi, B 10 1115546 + MIR 2333; DB 44339, ERX9058852, Iran, Isfahan province, Sarcheshmeh road, after Joshaqan-Kamoo toward Sarcheshmeh, W of Kashan to Isfahan road, 19 May 2014, A. Ebrahimi, MIR 2337; DB 44371, ERX9058853, Georgia, Chevi, Fluminis Thergi ( = Terek) vallis, 42°40’N, 44°36’E, 27 Jul 1988, M. A. Fischer et al. 8995, W1999-00640. — *S. parviflora* Jacq., DB32231, ERX9058854, [Iran], Azerbaijan orient, 14 Jun 1977, K. H. Rechinger, B 10 1015433. — *S. persepolitana* Boiss., DB 44269, ERX9058855, Iran, Bakhtiari, between Borujen and Lordegan, before Pol-e Kare, the east valley of Gerdebisheh village, 22 Apr 2006, M. Mirtadzadini, B 10 1115568 + MIR 2220; DB 44345, ERX9058857, Iran, East Azarbijan province, 15 km of Tabriz to Azarshahr, Esphahlan village, s.d., A. Ebrahimi, MIR 2345; DB 44295, ERX9058856, Iran, NW of Isfahan, near Delijan on clay hill, 20 May 2010, M. Mirtadzadini, B 10 1115541 + MIR 2218. — *S. purpurea* L., DB32194, ERX9058722, Germany, Brandenburg, 27 May 2013, E. Zippel, B 10 0612621. — *S. radiata* Fisch. ex Ledeb., DB6443, ERX9058858, Russia, S Siberia, Altay Republic, 23 Jul 2002, E. von Raab-Straube, B 10 0149455. — *S. radicosa* Boiss., DB33140, ERX9058860, Turkey, C6 Hatay, Amanos Mountains, 18 Oct 1988, H. Kehl, B 10 0478816; DB 44382, ERX9058862, Iran, Azerbaijan, Sabalan,, 4 Aug 2011, J. Noroozi, W2011-0010776; DB33145, ERX9058861, Iraq, Erbil (Kurdistan), 28 Jul 1957, K. H. Rechinger, B 10 1015464; DB33128, ERX9058859, Turkey, Bolkar Dalgari, 4 Aug 1992, E. von Raab-Straube, B 10 0842984. — *S. renzii* Rech. f., DB 44372, ERX9058863, Iran, Azarbaijan occidentalis, Chalil Kuh, in montibus supra Selvana, 1800-2600 m., 4 Jul 1974, J. Renz, W1980-0001085. — *S. rosea* Waldst. and Kit., DB33126, ERX9058864, Montenegro, Pluzin, 29 Jul 2019, R. Vogt, B 10 0346560. — *S. rupicola* Hausskn., DB32192, ERX9058723, Iran, Mowdere, Sultanabad, 19 Jun 1904, T. Strauss, B 10 1015410; DB 44324, ERX9058865, Iran, Bakhtiari, Between Shahrekord and Esfahan, Kuhe rokh, 12 Mar 1985, M. Mirtadzadini, B 10 1115543 + MIR 2302.—*S. songorica* (Kar. and Kir.) Lipsch. and Vassilcz., DB 44258, ERX9058866, Iran, Kerman, Sardu, Dehdivan Village, 24 Jun 2017, M. Mirtadzadini, B 10 1115558 + MIR 2247.—*S. tragopogonoides* Regel and Schmalh., DB 44255, ERX9058724, Afghanistan, S Salang Valley, about 10 km N Jabal us Diraj, 1450 m, 23 May 1968, H. Freitag, MSB-169799.—*S. virgata* DC., DB 44254, ERX9058725, Pakistan, Karakorum, Hunza- und Nagar-Gebiet, Daintar, 36°22’N, 74°09’E, 3000 m, 12 May 1905, F. Lobbichler, M.—*S. virgata* DC., DB33154, ERX9058867, Pakistan, Karakoram, 21 Aug 1960, O. Polunin, B 10 1015458. — *Takhtajaniantha austriaca* (Willd.) Zaika et al., DB 44198, ERX9058728, Russia, Ulyanovskaya oblast, 2 Aug 2006, N. S. Pavlov, MW0550563; DB 44199, ERX9058729, Russia, Saratovskaya oblast, 9 May 2008, E. A. Kireev, MW0550564; DB32199, ERX9058870, Italy, Prov. Trento, 29 May 1965, Damboldt, B 10 0304238; DB32182, ERX9058726, Austria, Burgenland, 5 May 2006, T. Barta, B 10 0477353; DB32183, ERX9058727, Hungary, Pilisborosjenö, Nagykevély, 13 Apr 1990, T. Barta, B 10 0343618; DB 44235, ERX9058730, Russia, West Sibiria, Altayskiy Kray, 14 July 2004, A. Zemmrich, GFW 40444. —*T. capito* (Maxim.) Zaika et al., DB 44206, ERX9058731, Mongolia, SW East-Gobi province, 1000-1100 m, 5 Aug 1989, I. A. Gubanov, MW0194396. — *T. crispa* (M. Bieb.) Zaika et al., DB 44203, ERX9058733, Ukraine, Crimea, near Faros, 4 Apr 2015, M. A. Zaika, MW; DB 44200, ERX9058732, Russia, Kyzyl-ordynskii region, 12 Jul 1929, S. Lipschitz, MW0891796. —*T. grubovii* (Lipsch.) E. Hatami et al., DB 44230, ERX9058868, Russia, Altai mountains, 23 Jul 2004, A. Zemmrich, GFW 39621. — *T. ikonnikovii* (Krasch. and Lipsch.) Zaika et al., DB 44204, ERX9058734, Mongolia, Bayan-Ulegeiskii region, 27 Jul 1988, A. A. Budanzev et al., MW0194381; DB 44215, ERX9058735, [Mongolia] 80 km NEE from town Altay, 1750 m, 6 Jul 1984, I. A. Gubanov, MW0194386. — *T. mongolica* (Maxim.) Zaika et al., DB33148, ERX9058869, China, Turkestania sinensis, in deserto Taklamakan, 22 May 1934, D. Hummel, B 10 0647061.—*T. pseudodivaricata* (Lipsch.) Zaika et al., DB 44208, ERX9058736, Mongolia, Gobian Altay, 6 Aug 1981, I. A. Gubanov, MW0194402; DB 44216, ERX9058737, Mongolia, Middle-Gobian region, 4 Jul 1979, Grubov, LE; DB 44243, ERX9058738, Mongolia, province of Khvod, Dörgön district, 29 Aug 2003, A. Zemmrich, GFW 44155. — *T. pusilla* (Pall.) Nazarova, DB 44210, ERX9058739, Kazakhstan, Sultan-Uiz-Dag mountains, 13 May 1934, Arsenieva, MW0892019. — *T. tau-saghyz* (Lipsch. and G. G. Bosse) Zaika et al., DB 44205, ERX9058740, Kazakhstan, Karatau mountains, 11 May 1939, N. V. Pavlov, MW0891885; DB 44219, ERX9058741, [Kazakhstan] Karatau, 12 Jun 1952, I. Paraonikova, MW0892087. — *Tragopogon crocifolius* L., DB32186, ERX9058742, Greece, Trikala, 23 May 2005, R. Willing and E. Willing, B 10 0211853.—*T. dubius* Scop., SRX5893216.
