# Supplementary figures and images for: New Insights Into the Relationships Within Subtribe Scorzonerinae (Cichorieae, Asteraceae) Using Hybrid Capture Phylogenomics (Hyb-Seq)
Source: Front Plant Sci. 2022 Jul 1;13:851716. doi: 10.3389/fpls.2022.851716 (PMC9298463; doi:10.3389/fpls.2022.851716)

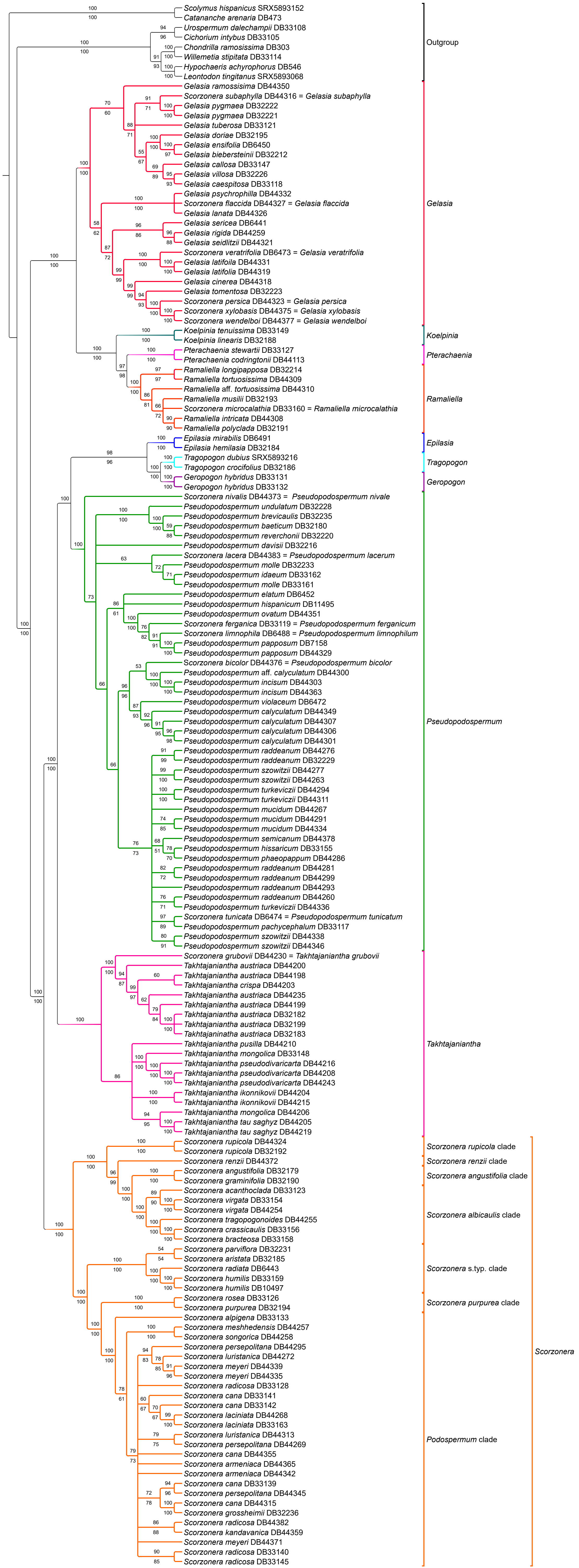

Supplement: Supplementary file 2 [file Image_2.pdf]

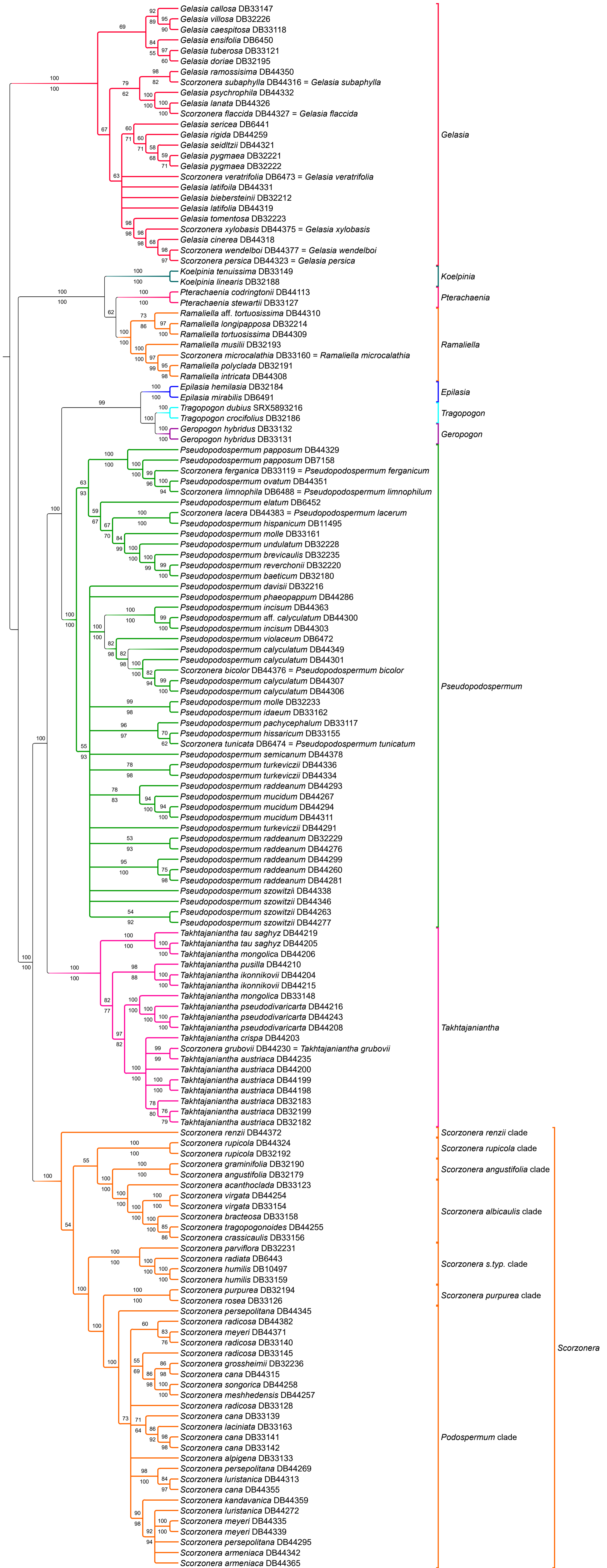

Supplement: Supplementary file 4 [file Image_4.pdf]

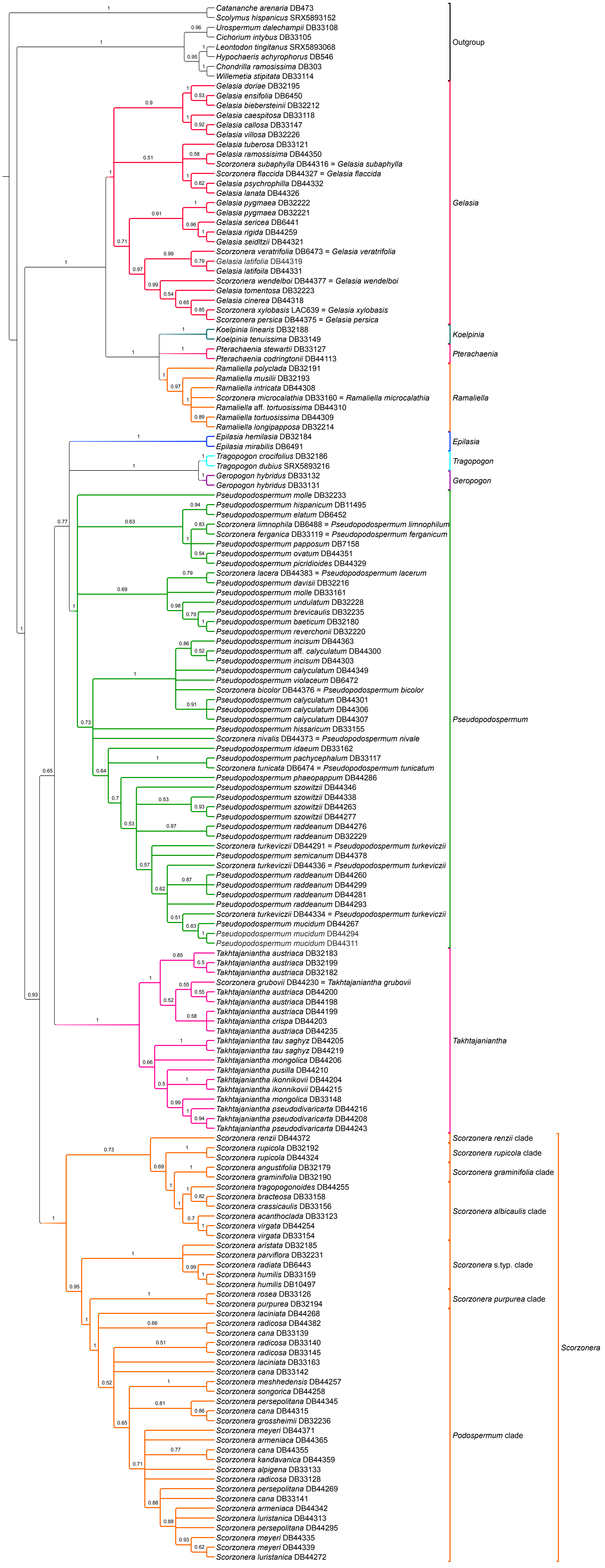

Supplement: Supplementary file 10 [file Data_Sheet_3.PDF]

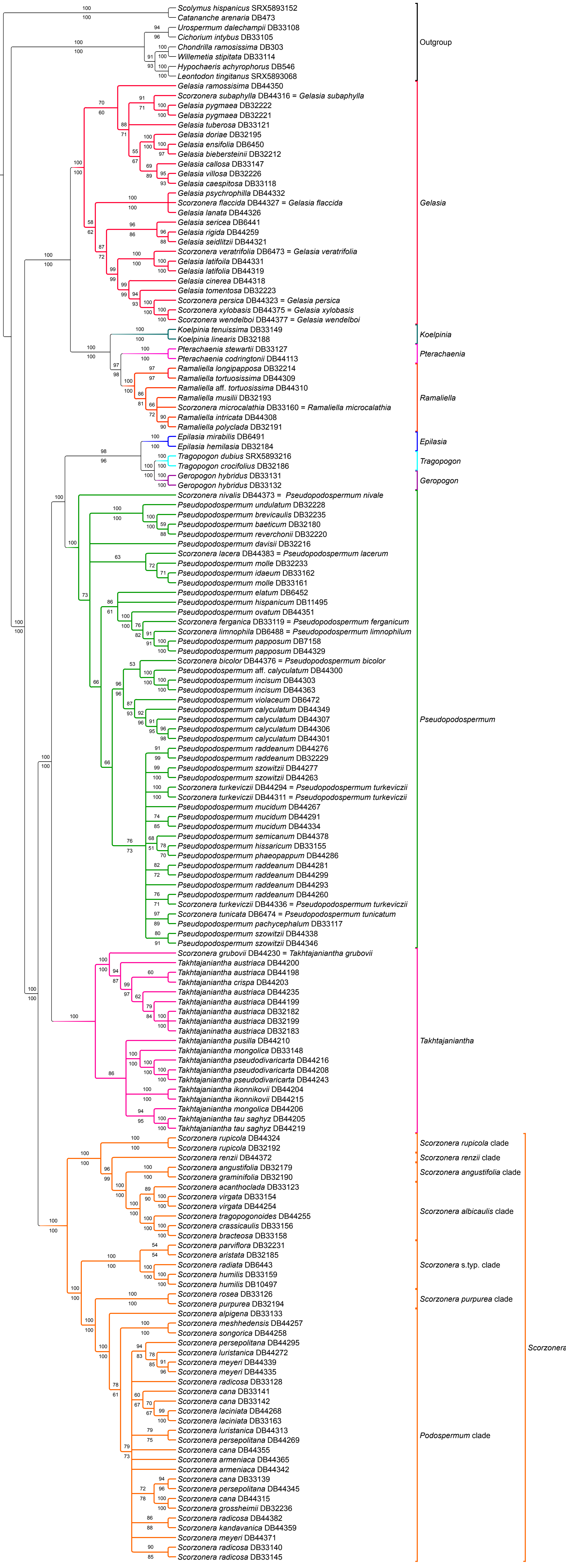

Supplement: Supplementary file 11 [file Data_Sheet_4.PDF]

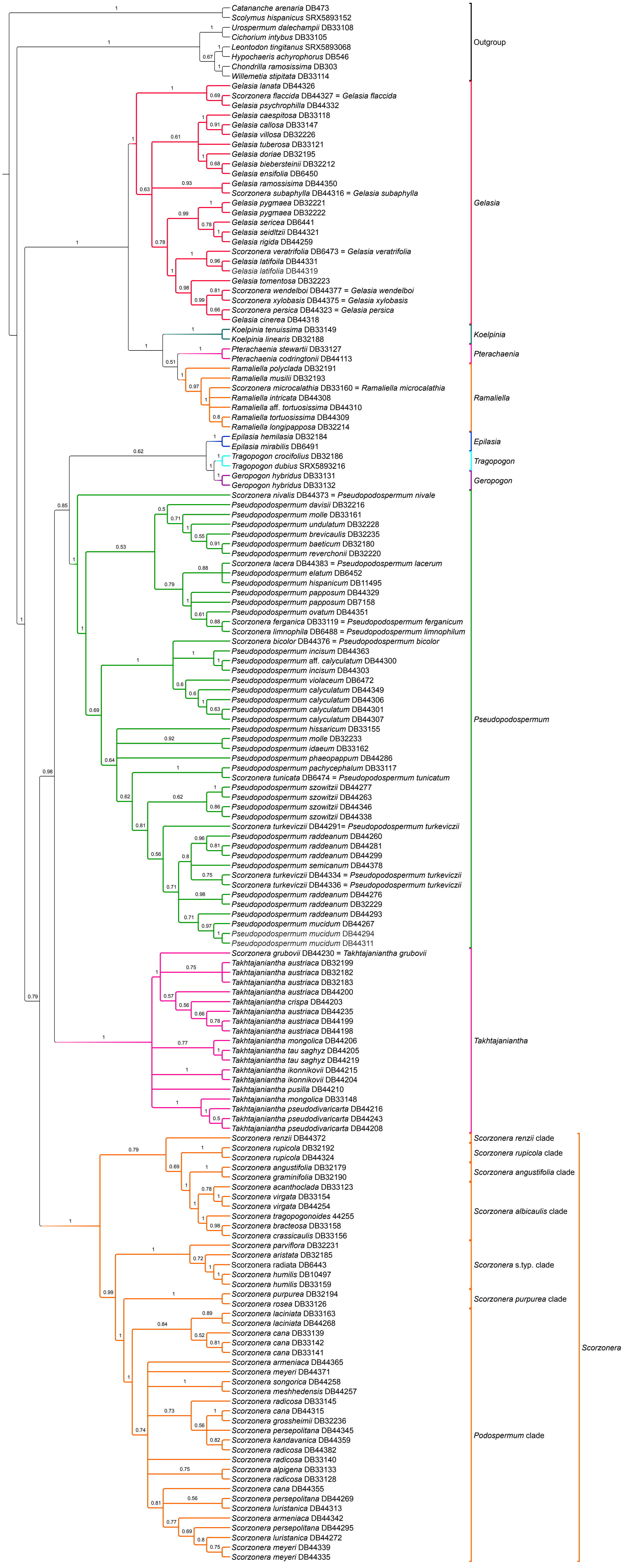

Supplement: Supplementary file 12 [file Data_Sheet_5.PDF]

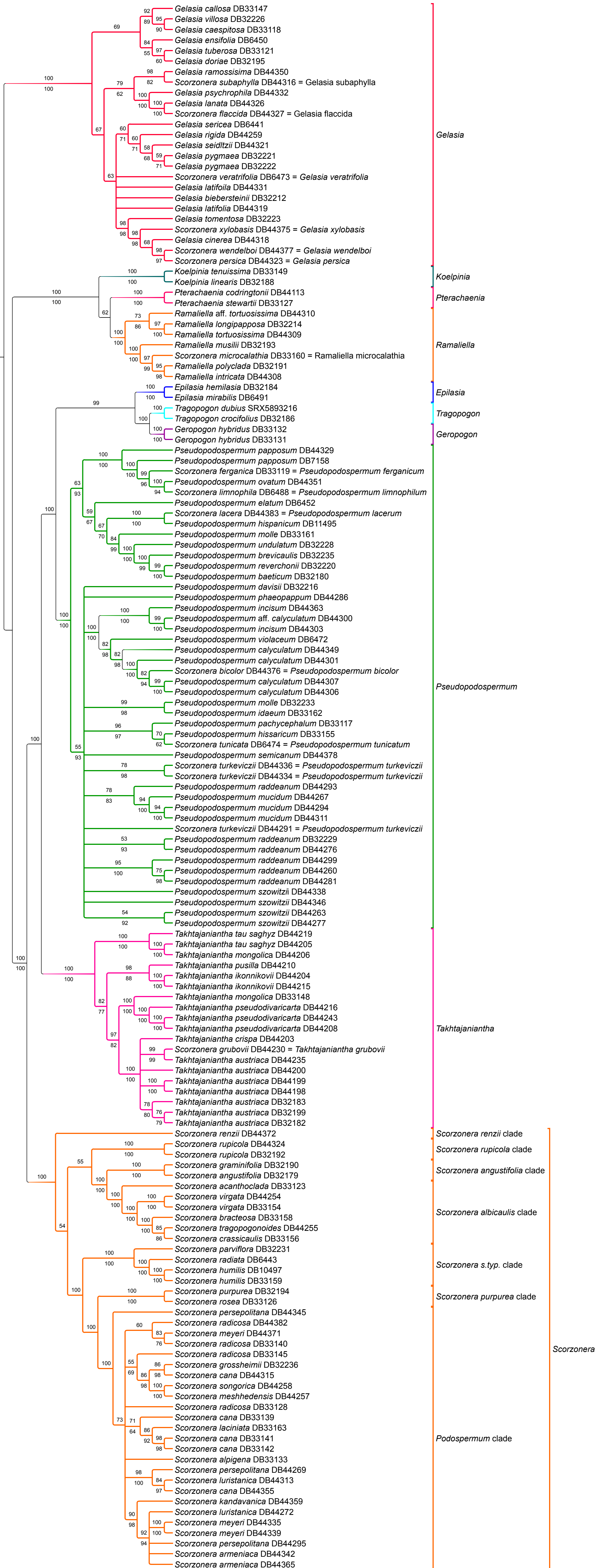

Supplement: Supplementary file 13 [file Data_Sheet_6.PDF]

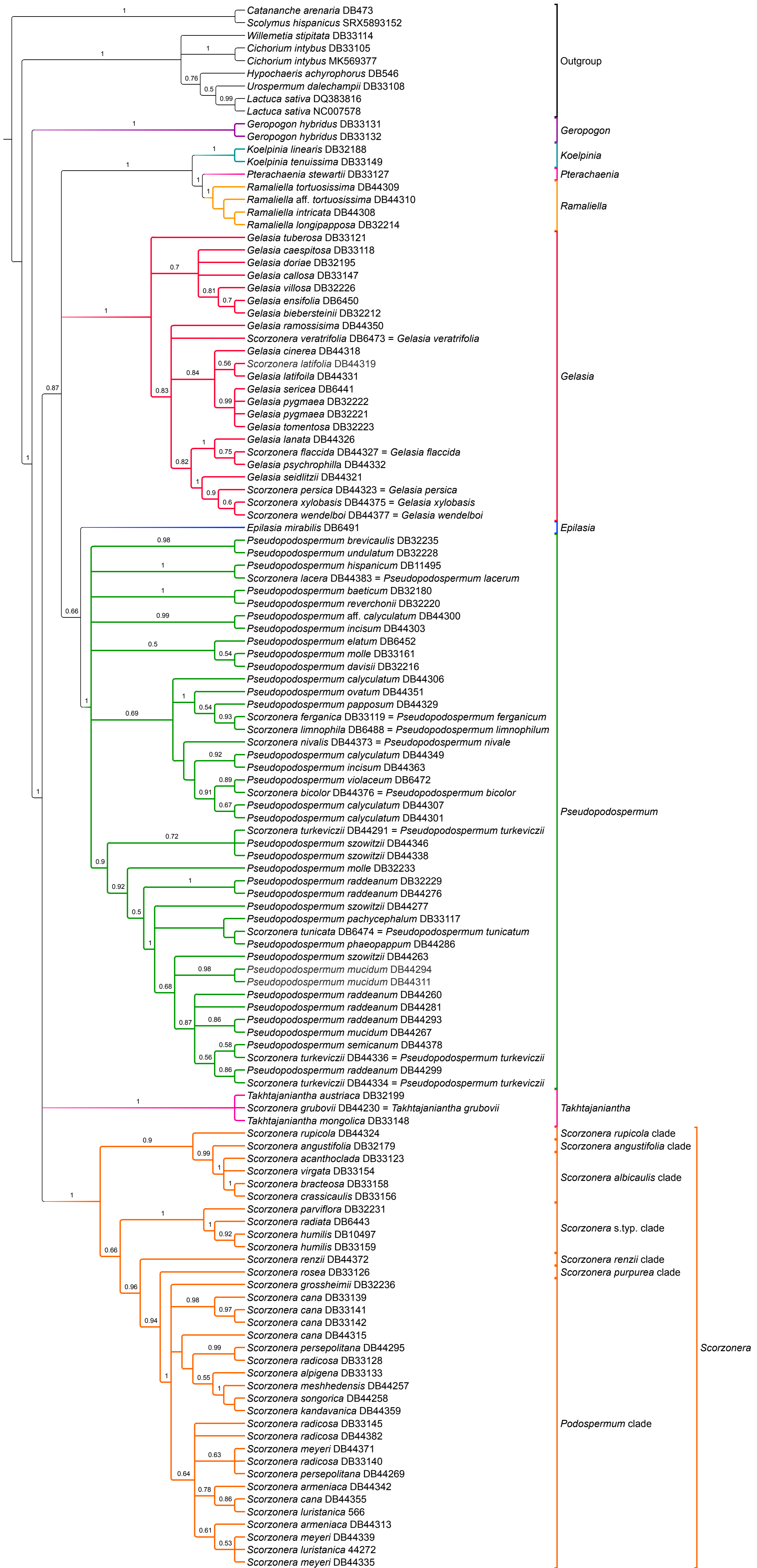

Supplement: Supplementary file 14 [file Data_Sheet_7.PDF]

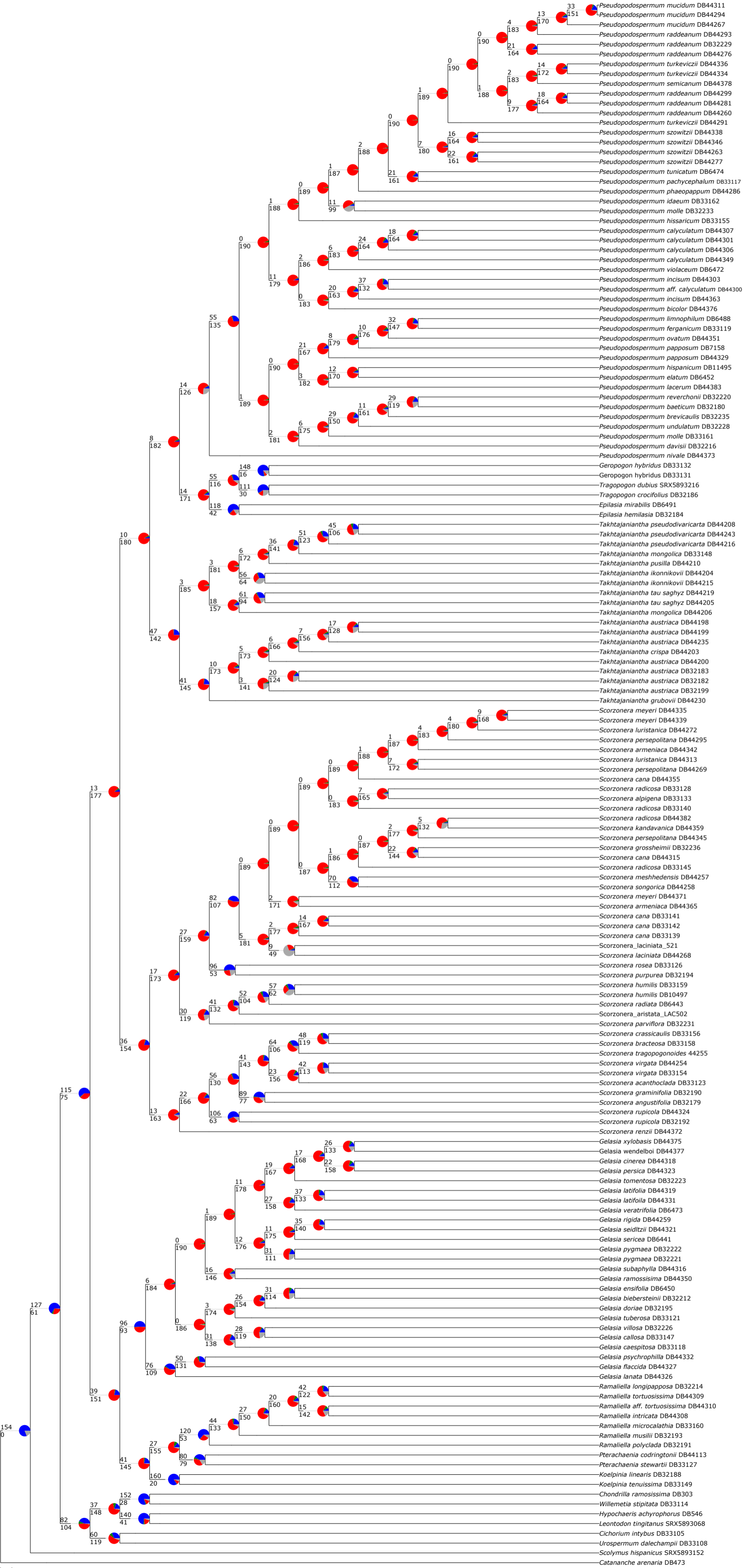

Supplement: Supplementary file 15 [file Data_Sheet_8.PDF]
