# Supplementary material for: New Insights Into the Relationships Within Subtribe Scorzonerinae (Cichorieae, Asteraceae) Using Hybrid Capture Phylogenomics (Hyb-Seq)
Source: Front Plant Sci. 2022 Jul 1;13:851716. doi: 10.3389/fpls.2022.851716 (PMC9298463; doi:10.3389/fpls.2022.851716)
Supplement: Supplementary file 3 [file Image_3.pdf]

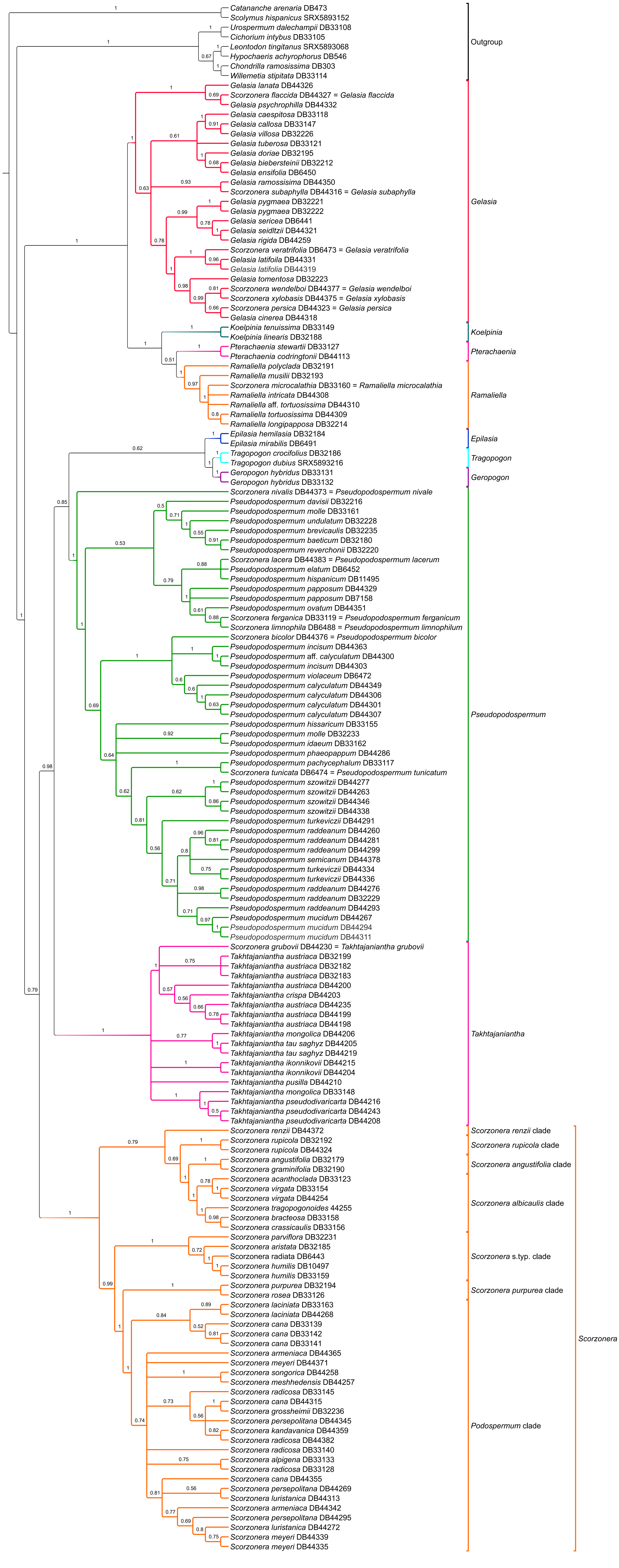

Supplementary Figure 3. Coalescent species tree of subtribe Scorzonerineae based on ELS dataset. Branch labels indicate support values of local posterior probabilities (LPP) and branches with less than 0.5 LPP are collapsed.
