# Supplementary material for: New Insights Into the Relationships Within Subtribe Scorzonerinae (Cichorieae, Asteraceae) Using Hybrid Capture Phylogenomics (Hyb-Seq)
Source: Front Plant Sci. 2022 Jul 1;13:851716. doi: 10.3389/fpls.2022.851716 (PMC9298463; doi:10.3389/fpls.2022.851716)
Supplement: Supplementary file 6 [file Image_6.pdf]

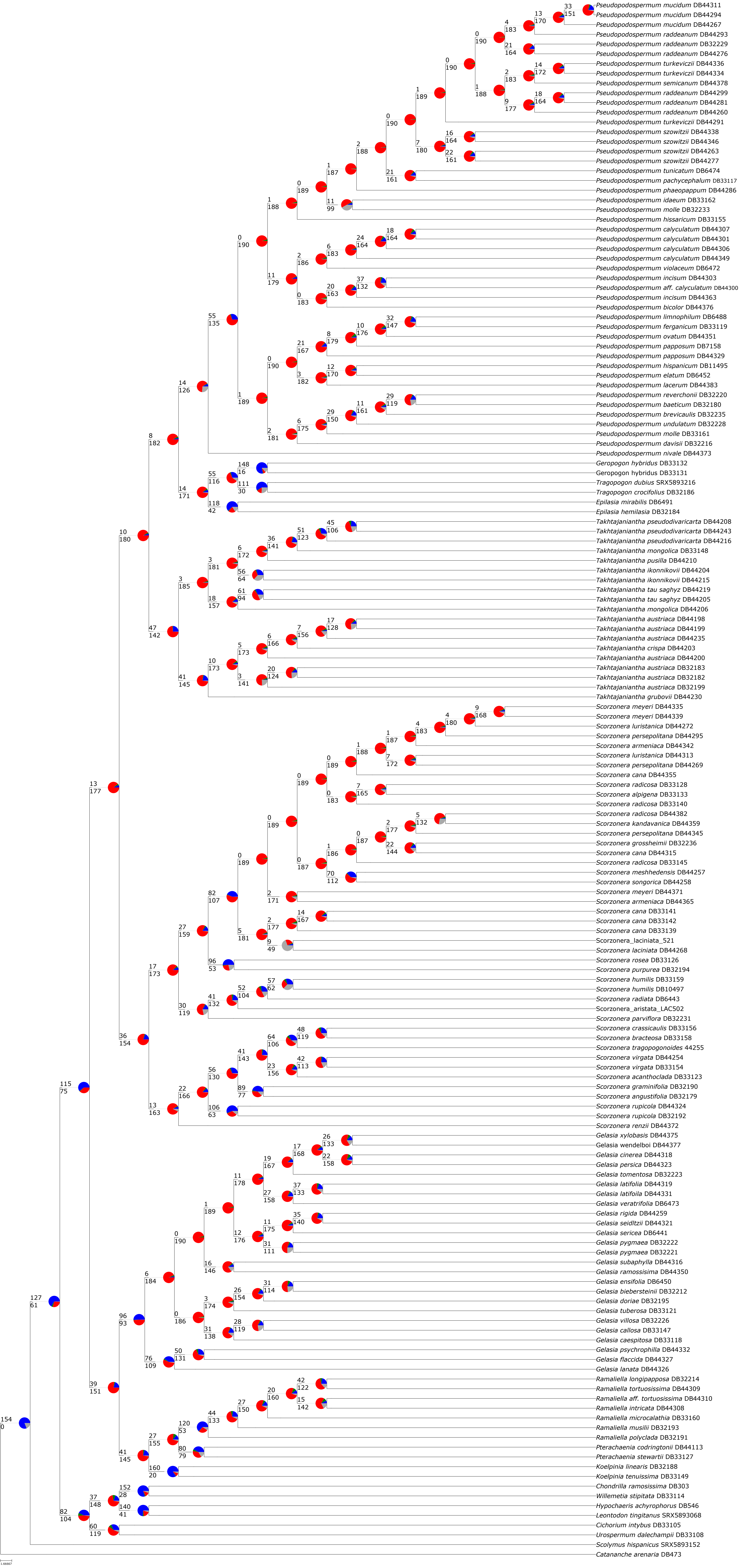

Supplementary Figure 6. ELS coalescent species tree with a summary of concordant and discordant gene trees. For each branch, the top number indicates the number of concordant gene trees and the bottom, the number of conflicting gene trees. The pie charts indicate the proportion of gene trees that support that clade (blue), the proportion that supports the main alternative for that clade (green), the proportion that supports all other topologies (red) or the proportion of uninformative gene trees for that clade (grey).
