# Supplementary material for: New Insights Into the Relationships Within Subtribe Scorzonerinae (Cichorieae, Asteraceae) Using Hybrid Capture Phylogenomics (Hyb-Seq)
Source: Front Plant Sci. 2022 Jul 1;13:851716. doi: 10.3389/fpls.2022.851716 (PMC9298463; doi:10.3389/fpls.2022.851716)
Supplement: Supplementary file 7 [file Table_1.pdf]

**Supplementary Table 1.** Statistics for the nuclear (OLS, ELS, supercontigs) datasets before removing loci with <50% of all samples, and plastome CDS dataset before removing loci with <50% of all samples and 10 parsimony informative sites.

| Dataset | Number of taxa | Number of recovered loci | Length of the concatenated matrix | Number/Percent of variable sites | Number/Percent of parsimony informative sites | Average percent of missing data per loci (min-max) | Average number of taxa recovered per loci (min-max) | Average sequence length per loci (min-max) |
|---|---|---|---|---|---|---|---|---|
| COS contigs (OLS) | 160 | 164 | 47,640 | 26,389/52% | 16,912/33% | 8.32% (0–27.52%) | 135 (1–160) | 290 (69–772) |
| COS contigs (ELS) | 160 | 234 | 68,339 | 37,579/52% | 24,351/34% | 8.27% (0–27.4%) | 136 (1–160) | 292 (69–773) |
| Supercon-tigs | 149 | 193 | 163,624 | 104,553/62 % | 74,994/44% | 7.18% (0–11.1%) | 84 (1–133) | 848 (424–1978) |
| Plastome CDS | 127 | 89 | 177,166 | 76,533/51% | 41,765/23% | 34.8% (0–93.9%) | 112 (1–127 | 1990 (78–24,594) |
