# Supplementary material for: New Insights Into the Relationships Within Subtribe Scorzonerinae (Cichorieae, Asteraceae) Using Hybrid Capture Phylogenomics (Hyb-Seq)
Source: Front Plant Sci. 2022 Jul 1;13:851716. doi: 10.3389/fpls.2022.851716 (PMC9298463; doi:10.3389/fpls.2022.851716)
Supplement: Supplementary file 8 [file Data_Sheet_1.pdf]

Data Sheet S1. Content of the Mesquite nexus file for ancestral character state reconstruction of pollen types in the Scorzonerinae using the nuclear ELS coalescent backbone tree as phylogenetic hypothesis.

```
#NEXUS
[written Fri Feb 11 16:42:55 CET 2022 by Mesquite  version 3.70 (build 
940) at BGBM13124/130.133.69.160]

BEGIN TAXA;
	TITLE Taxa;
	DIMENSIONS NTAX=17;
	TAXLABELS
		Epilasia_ Gelasia Geropogon_ Koelpinia_ Tourneuxia 
Podospermum_clade Pseudopodospermum Pterachaenia_ Ramaliella 
Scorzonera_albicaulis_clade Scorzonera_angustifolia_clade 
Scorzonera_purpurea_clade Scorzonera_renzii_clade 
Scorzonera_rupicola_clade Scorzonera_s.typ._clade Takhtajaniantha 
Tragopogon 
	;

END;


BEGIN CHARACTERS;
	TITLE  Character_Matrix;
	DIMENSIONS  NCHAR=1;
	FORMAT DATATYPE = STANDARD RESPECTCASE GAP = - MISSING = ? SYMBOLS 
= "  0 1 2 3 4 5 6";
	CHARSTATELABELS 
		1  /  '1-humilis' '2a-laciniata' '2b-hispanica' '2c-lanata' 
'3a-Tourneuxia' '3b-Tragopogon' '4-Epilasia' ; 
	MATRIX
	Epilasia_                     6
	Gelasia                       (1 3)
	Geropogon_                    3
	Koelpinia_                    5
	Tourneuxia                    4
	Podospermum_clade             (1 3)
	Pseudopodospermum             (1 2)
	Pterachaenia_                 (1 4)
	Ramaliella                    3
	Scorzonera_albicaulis_clade   3
	Scorzonera_angustifolia_clade 3
	Scorzonera_purpurea_clade     1
	Scorzonera_renzii_clade       3
	Scorzonera_rupicola_clade     ?
	Scorzonera_s.typ._clade       0
	Takhtajaniantha               0
	Tragopogon                    5

;

END;
BEGIN TREES;
	Title 'Trees from "ELS-coalAst.tre"';
	ID 017ee44c8f971;
	LINK Taxa = Taxa;
```

```
	TRANSLATE
[0]		n0 Epilasia_,
[1]		n1 Gelasia,
[2]		n2 Geropogon_,
[3]		n3 Koelpinia_,
[4]		n4 Tourneuxia,
[5]		n5 Podospermum_clade,
[6]		n6 Pseudopodospermum,
[7]		n7 Pterachaenia_,
[8]		n8 Ramaliella,
[9]		n9 Scorzonera_albicaulis_clade,
[10]		n10 Scorzonera_angustifolia_clade,
[11]		n11 Scorzonera_purpurea_clade,
[12]		n12 Scorzonera_renzii_clade,
[13]		n13 Scorzonera_rupicola_clade,
[14]		n14 Scorzonera_s.typ._clade,
[15]		n15 Takhtajaniantha,
[16]		n16 Tragopogon;
	TREE 'tree1+' =
(n4,((n1,(n3,(n7,n8):0.02803947916951041[%posteriorProbability = 0.51
]):0.26750807818594513[%posteriorProbability = 1.0
]):0.5205030895270601[%posteriorProbability = 1.0
],((((n0,(n16,n2):0.4587211453096614 6[%posteriorProbability = 1.0
]):0.04382356521887497[%posteriorProbability = 0.62
],n6):0.07894056974609316[%posteriorProbability = 0.85
],n15):0.13420071050218388[%posteriorProbability = 0.98
],((n12,(n13,(n10,n9):0.5861861125971851[%posteriorProbability = 1.0
]):0.1086261138192203[%posteriorProbability = 0.69
]):0.10768477625643676[%posteriorProbability = 0.79
],(n14,(n11,n5):0.269740658222234[%posteriorProbability = 1.0
]):0.1455608068679168[%posteriorProbability = 0.99
]):0.7860869141274511[%posteriorProbability = 1.0
]):0.0705384583631241[%posteriorProbability = 0.79
]):1.21512862731697[%posteriorProbability = 1.0 ]):0.33131713935438883[%
] [% ] [%  setBetweenDouble = posteriorProbability ];

END;


BEGIN ASSUMPTIONS;
	TYPESET * UNTITLED   =  unord:  1;

END;

BEGIN MESQUITECHARMODELS;
	ProbModelSet * UNTITLED   =  'Mk1 (est.)':  1;
END;

Begin MESQUITE;
		MESQUITESCRIPTVERSION 2;
		TITLE AUTO;
		tell ProjectCoordinator;
		timeSaved 1644594175511;
		getEmployee #mesquite.minimal.ManageTaxa.ManageTaxa;
		tell It;
			setID 0 2161254054645076160;
			tell It;
				setDefaultOrder  5 1 7 2 0 16 8 3 4 13 12 15 10 11
14 9 6;
				attachments ;
```

```
				endTell;
			endTell;
			getEmployee
#mesquite.charMatrices.ManageCharacters.ManageCharacters;
			tell It;
				setID 0 6087454669693722133;
				mqVersion 370;
				checksumv 0 3 1034496296 null  getNumChars 1 numChars 1
getNumTaxa 17 numTaxa 17   short true   bits 127   states 127
sumSquaresStatesOnly 7286.0 sumSquares 7286.0 longCompressibleToShort
false usingShortMatrix true   NumFiles 1 NumMatrices 1;
				mqVersion;
			endTell;
			getWindow;
			tell It;
				suppress;
				setResourcesState false false 306;
				setPopoutState 300;
				setExplanationSize 0;
				setAnnotationSize 0;
				setFontIncAnnot 0;
				setFontIncExp 0;
				setSize 1689 823;
				setLocation 118 47;
				setFont SanSerif;
				setFontSize 10;
				getToolPalette;
				tell It;
				endTell;
				desuppress;
			endTell;
			getEmployee  #mesquite.minimal.ManageTaxa.ManageTaxa;
			tell It;
				showTaxa #2161254054645076160
#mesquite.lists.TaxonList.TaxonList;
				tell It;
					setTaxa #2161254054645076160;
					getWindow;
					tell It;
						useTargetValue off;
						setTargetValue ;
						newAssistant
#mesquite.lists.TaxonListCurrPartition.TaxonListCurrPartition;
						setExplanationSize 30;
						setAnnotationSize 20;
						setFontIncAnnot 0;
						setFontIncExp 0;
						setSize 1383 751;
						setLocation 118 47;
						setFont SanSerif;
						setFontSize 10;
						getToolPalette;
						tell It;
							setTool
mesquite.lists.TaxonList.TaxonListWindow.ibeam;
						endTell;
					endTell;
					showWindow;
					getEmployee #mesquite.lists.ColorTaxon.ColorTaxon;
					tell It;
```

```
                              setColor Red;
                              removeColor off;
                        endTell;
                        getEmployee
#mesquite.lists.TaxonListAnnotPanel.TaxonListAnnotPanel;
                        tell It;
                              togglePanel off;
                        endTell;
                  endTell;
            endTell;
            getEmployee
#mesquite.trees.BasicTreeWindowCoord.BasicTreeWindowCoord;
            tell It;
                  makeTreeWindow #2161254054645076160
#mesquite.trees.BasicTreeWindowMaker.BasicTreeWindowMaker;
                  tell It;
                        suppressEPCResponse;
                        setTreeSource
#mesquite.trees.StoredTrees.StoredTrees;
                        tell It;
                              setTreeBlock 1;
                              setTreeBlockID 017ee44c8f971;
                              toggleUseWeights off;
                        endTell;
                        setAssignedID
1005.1644508420213.6778700437693428883;
                        getTreeWindow;
                        tell It;
                              setExplanationSize 30;
                              setAnnotationSize 20;
                              setFontIncAnnot 0;
                              setFontIncExp 0;
                              setSize 1383 751;
                              setLocation 118 47;
                              setFont SanSerif;
                              setFontSize 10;
                              getToolPalette;
                              tell It;
                                    setTool
mesquite.trees.ColorBranches.ColorToolExtra.ColorBranches;
                              endTell;
                              setActive;
                              getTreeDrawCoordinator
#mesquite.trees.BasicTreeDrawCoordinator.BasicTreeDrawCoordinator;
                        tell It;
                              suppress;
                              setTreeDrawer
#mesquite.trees.SquareLineTree.SquareLineTree;
                              tell It;
                                    setNodeLocs
#mesquite.trees.NodeLocsStandard.NodeLocsStandard;
                                    tell It;
                                          branchLengthsToggle off;
                                          toggleScale on;
                                          toggleBroadScale off;
                                          toggleCenter on;
                                          toggleEven on;
                                          setFixedTaxonDistance 0;
                                    endTell;
                                    setEdgeWidth 12;
```

```
					showEdgeLines on;
					orientRight;
				endTell;
				setBackground White;
				setBranchColor Black;
				showNodeNumbers off;
				showBranchColors on;
				labelBranchLengths off;
				centerBrLenLabels on;
				showBrLensUnspecified on;
				showBrLenLabelsOnTerminals on;
				setBrLenLabelColor 0 0 255;
				setNumBrLenDecimals 6;
				setSelectedTaxonHighlightMode 1;
				desuppress;
				getEmployee
#mesquite.trees.BasicDrawTaxonNames.BasicDrawTaxonNames;
				tell It;
					setFontSize 12;
					setColor Black;
					setTaxonNameStyler
#mesquite.trees.NoColorForTaxon.NoColorForTaxon;
					toggleShadePartition off;
					toggleShowFootnotes on;
					toggleNodeLabels on;
					toggleCenterNodeNames off;
					toggleShowNames on;
					namesAngle ?;
				endTell;
			endTell;
				setTreeNumber 1;
				setDrawingSizeMode 0;
				toggleLegendFloat on;
				scale 0;
				toggleTextOnTree off;
				togglePrintName off;
				showWindow;
				newAssistant
#mesquite.ancstates.TraceCharacterHistory.TraceCharacterHistory;
			tell It;
				suspend ;
				setDisplayMode
#mesquite.ancstates.ShadeStatesOnTree.ShadeStatesOnTree;
				tell It;
					toggleLabels off;
					togglePredictions off;
					toggleGray off;
				endTell;
				setHistorySource
#mesquite.ancstates.RecAncestralStates.RecAncestralStates;
				tell It;
					getCharacterSource
#mesquite.charMatrices.CharSrcCoordObed.CharSrcCoordObed;
					tell It;
						setCharacterSource
#mesquite.charMatrices.StoredCharacters.StoredCharacters;
						tell It;
							setDataSet
#6087454669693722133;
						endTell;
```

```
                                endTell;
                                setMethod
#mesquite.parsimony.ParsAncestralStates.ParsAncestralStates;
                                tell It;
                                     setModelSource
#mesquite.parsimony.CurrentParsModels.CurrentParsModels;
                                     toggleMPRsMode off;
                                endTell;
                                toggleShowSelectedOnly off;
                           endTell;
                           setCharacter 1;
                           setMapping 1;
                           toggleShowLegend on;
                           setColorMode 0;
                           toggleWeights on;
                           setInitialOffsetX 3;
                           setInitialOffsetY 440;
                           setLegendWidth 142;
                           setLegendHeight 295;
                           resume ;
                      endTell;
                           newAssistant
#mesquite.ancstates.TraceCharacterHistory.TraceCharacterHistory;
                      tell It;
                           suspend ;
                           setDisplayMode
#mesquite.ancstates.ShadeStatesOnTree.ShadeStatesOnTree;
                           tell It;
                                toggleLabels off;
                                togglePredictions off;
                                toggleGray off;
                           endTell;
                           setHistorySource
#mesquite.ancstates.RecAncestralStates.RecAncestralStates;
                           tell It;
                                getCharacterSource
#mesquite.charMatrices.CharSrcCoordObed.CharSrcCoordObed;
                                tell It;
                                     setCharacterSource
#mesquite.charMatrices.StoredCharacters.StoredCharacters;
                                     tell It;
                                          setDataSet
#6087454669693722133;
                                     endTell;
                                endTell;
                                setMethod
#mesquite.parsimony.ParsAncestralStates.ParsAncestralStates;
                                tell It;
                                     setModelSource
#mesquite.parsimony.CurrentParsModels.CurrentParsModels;
                                     toggleMPRsMode off;
                                endTell;
                                toggleShowSelectedOnly off;
                           endTell;
                           setCharacter 1;
                           setMapping 1;
                           toggleShowLegend on;
                           setColorMode 0;
                           toggleWeights on;
                           setInitialOffsetX 2;
```

```
                              setInitialOffsetY 440;
                              setLegendWidth 142;
                              setLegendHeight 295;
                              resume ;
                              modifyColors  6 0  38 20 245;
                         endTell;
                         endTell;
                         desuppressEPCResponse;
                         getEmployee
#mesquite.trees.ColorBranches.ColorBranches;
                         tell It;
                              setColor Red;
                              removeColor off;
                         endTell;
                         getEmployee
#mesquite.ornamental.BranchNotes.BranchNotes;
                         tell It;
                              setAlwaysOn off;
                         endTell;
                         getEmployee
#mesquite.ornamental.ColorTreeByPartition.ColorTreeByPartition;
                         tell It;
                              colorByPartition off;
                         endTell;
                         getEmployee
#mesquite.ornamental.DrawTreeAssocDoubles.DrawTreeAssocDoubles;
                         tell It;
                              setOn on;
                              toggleShow consensusFrequency;
                              toggleShow posteriorProbability;
                              toggleShow bootstrapFrequency;
                              toggleShow consensusFrequency;
                              toggleShow posteriorProbability;
                              toggleShow bootstrapFrequency;
                              setDigits 4;
                              setThreshold ?;
                              writeAsPercentage off;
                              toggleCentred off;
                              toggleHorizontal on;
                              toggleWhiteEdges off;
                              toggleShowOnTerminals off;
                              setFontSize 2;
                              setOffset 0  0;
                         endTell;
                         getEmployee
#mesquite.ornamental.DrawTreeAssocStrings.DrawTreeAssocStrings;
                         tell It;
                              setOn on;
                              toggleCentred off;
                              toggleHorizontal on;
                              setFontSize 10;
                              setOffset 0  0;
                              toggleShowOnTerminals off;
                         endTell;
                         getEmployee
#mesquite.trees.TreeInfoValues.TreeInfoValues;
                         tell It;
                              panelOpen false;
                         endTell;
                    endTell;
```

```
			endTell;
			getEmployee
#mesquite.charMatrices.BasicDataWindowCoord.BasicDataWindowCoord;
			tell It;
				showDataWindow #6087454669693722133
#mesquite.charMatrices.BasicDataWindowMaker.BasicDataWindowMaker;
				tell It;
					getWindow;
					tell It;
						setExplanationSize 30;
						setAnnotationSize 20;
						setFontIncAnnot 0;
						setFontIncExp 0;
						setSize 1383 751;
						setLocation 118 47;
						setFont SanSerif;
						setFontSize 10;
						getToolPalette;
						tell It;
							setTool
mesquite.charMatrices.ColorCells.ColorCells.ColorCells;
						endTell;
						setTool
mesquite.charMatrices.ColorCells.ColorCells.ColorCells;
						colorCells
#mesquite.charMatrices.NoColor.NoColor;
						colorRowNames
#mesquite.charMatrices.TaxonGroupColor.TaxonGroupColor;
						colorColumnNames
#mesquite.charMatrices.CharGroupColor.CharGroupColor;
						colorText
#mesquite.charMatrices.NoColor.NoColor;
						setBackground White;
						toggleShowNames on;
						toggleShowTaxonNames on;
						toggleTight off;
						toggleThinRows off;
						toggleShowChanges on;
						toggleSeparateLines off;
						toggleShowStates on;
						toggleReduceCellBorders off;
						toggleAutoWCharNames on;
						toggleAutoTaxonNames off;
						toggleShowDefaultCharNames off;
						toggleConstrainCW on;
						toggleBirdsEye off;
						toggleColorOnlyTaxonNames off;
						toggleShowPaleGrid off;
						toggleShowPaleCellColors off;
						toggleShowPaleExcluded off;
						togglePaleInapplicable on;
						togglePaleMissing off;
						toggleShowBoldCellText off;
						toggleAllowAutosize on;
						toggleColorsPanel off;
						toggleDiagonal on;
						setDiagonalHeight 80;
						toggleLinkedScrolling on;
						toggleScrollLinkedTables off;
					endTell;
```

```
                    showWindow;
                    getWindow;
                    tell It;
                         forceAutosize;
                    endTell;
                    getEmployee
#mesquite.charMatrices.AlterData.AlterData;
                    tell It;
                         toggleBySubmenus off;
                    endTell;
                    getEmployee
#mesquite.charMatrices.ColorByState.ColorByState;
                    tell It;
                         setStateLimit 9;
                         toggleUniformMaximum on;
                    endTell;
                    getEmployee
#mesquite.charMatrices.ColorCells.ColorCells;
                    tell It;
                         setColor Red;
                         removeColor off;
                    endTell;
                    getEmployee
#mesquite.categ.StateNamesEditor.StateNamesEditor;
                    tell It;
                         makeWindow;
                         tell It;
                              setExplanationSize 30;
                              setAnnotationSize 20;
                              setFontIncAnnot 0;
                              setFontIncExp 0;
                              setSize 1383 751;
                              setLocation 118 47;
                              setFont SanSerif;
                              setFontSize 10;
                              getToolPalette;
                              tell It;
                                   setTool
mesquite.categ.StateNamesEditor.StateNamesWindow.ibeam;
                              endTell;
                              rowsAreCharacters on;
                              toggleConstrainChar on;
                              toggleConstrainCharNum 3;
                              togglePanel off;
                              toggleSummaryPanel off;
                         endTell;
                         showWindow;
                    endTell;
                    getEmployee
#mesquite.categ.StateNamesStrip.StateNamesStrip;
                    tell It;
                         showStrip off;
                    endTell;
                    getEmployee
#mesquite.charMatrices.AnnotPanel.AnnotPanel;
                    tell It;
                         togglePanel off;
                    endTell;
                    getEmployee
#mesquite.charMatrices.CharReferenceStrip.CharReferenceStrip;
```

```
                        tell It;
                              showStrip off;
                        endTell;
                        getEmployee
#mesquite.charMatrices.QuickKeySelector.QuickKeySelector;
                        tell It;
                              autotabOff;
                        endTell;
                        getEmployee
#mesquite.charMatrices.SelSummaryStrip.SelSummaryStrip;
                        tell It;
                              showStrip off;
                        endTell;
                        getEmployee
#mesquite.categ.SmallStateNamesEditor.SmallStateNamesEditor;
                        tell It;
                              panelOpen true;
                        endTell;
                  endTell;
            endTell;
            endTell;
end;
```
