# Supplementary material for: New Insights Into the Relationships Within Subtribe Scorzonerinae (Cichorieae, Asteraceae) Using Hybrid Capture Phylogenomics (Hyb-Seq)
Source: Front Plant Sci. 2022 Jul 1;13:851716. doi: 10.3389/fpls.2022.851716 (PMC9298463; doi:10.3389/fpls.2022.851716)
Supplement: Supplementary file 9 [file Data_Sheet_2.DOCX]

#NEXUS

[written Fri Feb 11 16:42:55 CET 2022 by Mesquite version 3.70 (build 940) at BGBM13124/130.133.69.160]

BEGIN TAXA;

TITLE Taxa;

DIMENSIONS NTAX=17;

TAXLABELS

Epilasia_ Gelasia Geropogon_ Koelpinia_ Tourneuxia Podospermum_clade Pseudopodospermum Pterachaenia_ Ramaliella Scorzonera_albicaulis_clade Scorzonera_angustifolia_clade Scorzonera_purpurea_clade Scorzonera_renzii_clade Scorzonera_rupicola_clade Scorzonera_s.typ._clade Takhtajaniantha Tragopogon

;

END;

BEGIN CHARACTERS;

TITLE Character_Matrix;

DIMENSIONS NCHAR=1;

FORMAT DATATYPE = STANDARD RESPECTCASE GAP = - MISSING = ? SYMBOLS = " 0 1 2 3 4 5 6";

CHARSTATELABELS

1 / '1-humilis' '2a-laciniata' '2b-hispanica' '2c-lanata' '3a-Tourneuxia' '3b-Tragopogon' '4-Epilasia' ;

MATRIX

Epilasia_ 6

Gelasia (1 3)

Geropogon_ 3

Koelpinia_ 5

Tourneuxia 4

Podospermum_clade (1 3)

Pseudopodospermum (1 2)

Pterachaenia_ (1 4)

Ramaliella 3

Scorzonera_albicaulis_clade 3

Scorzonera_angustifolia_clade 3

Scorzonera_purpurea_clade 1

Scorzonera_renzii_clade 3

Scorzonera_rupicola_clade ?

Scorzonera_s.typ._clade 0

Takhtajaniantha 0

Tragopogon 5

;

END;

BEGIN TREES;

Title 'Trees from "ELS-coalAst.tre"';

ID 017ee44c8f971;

LINK Taxa = Taxa;

TRANSLATE

[0] n0 Epilasia_,

[1] n1 Gelasia,

[2] n2 Geropogon_,

[3] n3 Koelpinia_,

[4] n4 Tourneuxia,

[5] n5 Podospermum_clade,

[6] n6 Pseudopodospermum,

[7] n7 Pterachaenia_,

[8] n8 Ramaliella,

[9] n9 Scorzonera_albicaulis_clade,

[10] n10 Scorzonera_angustifolia_clade,

[11] n11 Scorzonera_purpurea_clade,

[12] n12 Scorzonera_renzii_clade,

[13] n13 Scorzonera_rupicola_clade,

[14] n14 Scorzonera_s.typ._clade,

[15] n15 Takhtajaniantha,

[16] n16 Tragopogon;

TREE 'tree1+' = (n4,((n1,(n3,(n7,n8):0.02803947916951041[%posteriorProbability = 0.51 ]):0.26750807818594513[%posteriorProbability = 1.0 ]):0.5205030895270601[%posteriorProbability = 1.0 ],((((n0,(n16,n2):0.45872114530966146[%posteriorProbability = 1.0 ]):0.04382356521887497[%posteriorProbability = 0.62 ],n6):0.07894056974609316[%posteriorProbability = 0.85 ],n15):0.13420071050218388[%posteriorProbability = 0.98 ],((n12,(n13,(n10,n9):0.5861861125971851[%posteriorProbability = 1.0 ]):0.1086261138192203[%posteriorProbability = 0.69 ]):0.10768477625643676[%posteriorProbability = 0.79 ],(n14,(n11,n5):0.269740658222234[%posteriorProbability = 1.0 ]):0.1455608068679168[%posteriorProbability = 0.99 ]):0.7860869141274511[%posteriorProbability = 1.0 ]):0.0705384583631241[%posteriorProbability = 0.79 ]):1.21512862731697[%posteriorProbability = 1.0 ]):0.33131713935438883[% ] [% ] [% setBetweenDouble = posteriorProbability ];

END;

BEGIN ASSUMPTIONS;

TYPESET * UNTITLED = unord: 1;

END;

BEGIN MESQUITECHARMODELS;

ProbModelSet * UNTITLED = 'Mk1 (est.)': 1;

END;

Begin MESQUITE;

MESQUITESCRIPTVERSION 2;

TITLE AUTO;

tell ProjectCoordinator;

timeSaved 1644594175511;

getEmployee #mesquite.minimal.ManageTaxa.ManageTaxa;

tell It;

setID 0 2161254054645076160;

tell It;

setDefaultOrder 5 1 7 2 0 16 8 3 4 13 12 15 10 11 14 9 6;

attachments ;

endTell;

endTell;

getEmployee #mesquite.charMatrices.ManageCharacters.ManageCharacters;

tell It;

setID 0 6087454669693722133;

mqVersion 370;

checksumv 0 3 1034496296 null getNumChars 1 numChars 1 getNumTaxa 17 numTaxa 17 short true bits 127 states 127 sumSquaresStatesOnly 7286.0 sumSquares 7286.0 longCompressibleToShort false usingShortMatrix true NumFiles 1 NumMatrices 1;

mqVersion;

endTell;

getWindow;

tell It;

suppress;

setResourcesState false false 306;

setPopoutState 300;

setExplanationSize 0;

setAnnotationSize 0;

setFontIncAnnot 0;

setFontIncExp 0;

setSize 1689 823;

setLocation 118 47;

setFont SanSerif;

setFontSize 10;

getToolPalette;

tell It;

endTell;

desuppress;

endTell;

getEmployee #mesquite.minimal.ManageTaxa.ManageTaxa;

tell It;

showTaxa #2161254054645076160 #mesquite.lists.TaxonList.TaxonList;

tell It;

setTaxa #2161254054645076160;

getWindow;

tell It;

useTargetValue off;

setTargetValue ;

newAssistant #mesquite.lists.TaxonListCurrPartition.TaxonListCurrPartition;

setExplanationSize 30;

setAnnotationSize 20;

setFontIncAnnot 0;

setFontIncExp 0;

setSize 1383 751;

setLocation 118 47;

setFont SanSerif;

setFontSize 10;

getToolPalette;

tell It;

setTool mesquite.lists.TaxonList.TaxonListWindow.ibeam;

endTell;

endTell;

showWindow;

getEmployee #mesquite.lists.ColorTaxon.ColorTaxon;

tell It;

setColor Red;

removeColor off;

endTell;

getEmployee #mesquite.lists.TaxonListAnnotPanel.TaxonListAnnotPanel;

tell It;

togglePanel off;

endTell;

endTell;

endTell;

getEmployee #mesquite.trees.BasicTreeWindowCoord.BasicTreeWindowCoord;

tell It;

makeTreeWindow #2161254054645076160 #mesquite.trees.BasicTreeWindowMaker.BasicTreeWindowMaker;

tell It;

suppressEPCResponse;

setTreeSource #mesquite.trees.StoredTrees.StoredTrees;

tell It;

setTreeBlock 1;

setTreeBlockID 017ee44c8f971;

toggleUseWeights off;

endTell;

setAssignedID 1005.1644508420213.6778700437693428883;

getTreeWindow;

tell It;

setExplanationSize 30;

setAnnotationSize 20;

setFontIncAnnot 0;

setFontIncExp 0;

setSize 1383 751;

setLocation 118 47;

setFont SanSerif;

setFontSize 10;

getToolPalette;

tell It;

setTool mesquite.trees.ColorBranches.ColorToolExtra.ColorBranches;

endTell;

setActive;

getTreeDrawCoordinator #mesquite.trees.BasicTreeDrawCoordinator.BasicTreeDrawCoordinator;

tell It;

suppress;

setTreeDrawer #mesquite.trees.SquareLineTree.SquareLineTree;

tell It;

setNodeLocs #mesquite.trees.NodeLocsStandard.NodeLocsStandard;

tell It;

branchLengthsToggle off;

toggleScale on;

toggleBroadScale off;

toggleCenter on;

toggleEven on;

setFixedTaxonDistance 0;

endTell;

setEdgeWidth 12;

showEdgeLines on;

orientRight;

endTell;

setBackground White;

setBranchColor Black;

showNodeNumbers off;

showBranchColors on;

labelBranchLengths off;

centerBrLenLabels on;

showBrLensUnspecified on;

showBrLenLabelsOnTerminals on;

setBrLenLabelColor 0 0 255;

setNumBrLenDecimals 6;

setSelectedTaxonHighlightMode 1;

desuppress;

getEmployee #mesquite.trees.BasicDrawTaxonNames.BasicDrawTaxonNames;

tell It;

setFontSize 12;

setColor Black;

setTaxonNameStyler #mesquite.trees.NoColorForTaxon.NoColorForTaxon;

toggleShadePartition off;

toggleShowFootnotes on;

toggleNodeLabels on;

toggleCenterNodeNames off;

toggleShowNames on;

namesAngle ?;

endTell;

endTell;

setTreeNumber 1;

setDrawingSizeMode 0;

toggleLegendFloat on;

scale 0;

toggleTextOnTree off;

togglePrintName off;

showWindow;

newAssistant #mesquite.ancstates.TraceCharacterHistory.TraceCharacterHistory;

tell It;

suspend ;

setDisplayMode #mesquite.ancstates.ShadeStatesOnTree.ShadeStatesOnTree;

tell It;

toggleLabels off;

togglePredictions off;

toggleGray off;

endTell;

setHistorySource #mesquite.ancstates.RecAncestralStates.RecAncestralStates;

tell It;

getCharacterSource #mesquite.charMatrices.CharSrcCoordObed.CharSrcCoordObed;

tell It;

setCharacterSource #mesquite.charMatrices.StoredCharacters.StoredCharacters;

tell It;

setDataSet #6087454669693722133;

endTell;

endTell;

setMethod #mesquite.parsimony.ParsAncestralStates.ParsAncestralStates;

tell It;

setModelSource #mesquite.parsimony.CurrentParsModels.CurrentParsModels;

toggleMPRsMode off;

endTell;

toggleShowSelectedOnly off;

endTell;

setCharacter 1;

setMapping 1;

toggleShowLegend on;

setColorMode 0;

toggleWeights on;

setInitialOffsetX 3;

setInitialOffsetY 440;

setLegendWidth 142;

setLegendHeight 295;

resume ;

endTell;

newAssistant #mesquite.ancstates.TraceCharacterHistory.TraceCharacterHistory;

tell It;

suspend ;

setDisplayMode #mesquite.ancstates.ShadeStatesOnTree.ShadeStatesOnTree;

tell It;

toggleLabels off;

togglePredictions off;

toggleGray off;

endTell;

setHistorySource #mesquite.ancstates.RecAncestralStates.RecAncestralStates;

tell It;

getCharacterSource #mesquite.charMatrices.CharSrcCoordObed.CharSrcCoordObed;

tell It;

setCharacterSource #mesquite.charMatrices.StoredCharacters.StoredCharacters;

tell It;

setDataSet #6087454669693722133;

endTell;

endTell;

setMethod #mesquite.parsimony.ParsAncestralStates.ParsAncestralStates;

tell It;

setModelSource #mesquite.parsimony.CurrentParsModels.CurrentParsModels;

toggleMPRsMode off;

endTell;

toggleShowSelectedOnly off;

endTell;

setCharacter 1;

setMapping 1;

toggleShowLegend on;

setColorMode 0;

toggleWeights on;

setInitialOffsetX 2;

setInitialOffsetY 440;

setLegendWidth 142;

setLegendHeight 295;

resume ;

modifyColors 6 0 38 20 245;

endTell;

endTell;

desuppressEPCResponse;

getEmployee #mesquite.trees.ColorBranches.ColorBranches;

tell It;

setColor Red;

removeColor off;

endTell;

getEmployee #mesquite.ornamental.BranchNotes.BranchNotes;

tell It;

setAlwaysOn off;

endTell;

getEmployee #mesquite.ornamental.ColorTreeByPartition.ColorTreeByPartition;

tell It;

colorByPartition off;

endTell;

getEmployee #mesquite.ornamental.DrawTreeAssocDoubles.DrawTreeAssocDoubles;

tell It;

setOn on;

toggleShow consensusFrequency;

toggleShow posteriorProbability;

toggleShow bootstrapFrequency;

toggleShow consensusFrequency;

toggleShow posteriorProbability;

toggleShow bootstrapFrequency;

setDigits 4;

setThreshold ?;

writeAsPercentage off;

toggleCentred off;

toggleHorizontal on;

toggleWhiteEdges off;

toggleShowOnTerminals off;

setFontSize 2;

setOffset 0 0;

endTell;

getEmployee #mesquite.ornamental.DrawTreeAssocStrings.DrawTreeAssocStrings;

tell It;

setOn on;

toggleCentred off;

toggleHorizontal on;

setFontSize 10;

setOffset 0 0;

toggleShowOnTerminals off;

endTell;

getEmployee #mesquite.trees.TreeInfoValues.TreeInfoValues;

tell It;

panelOpen false;

endTell;

endTell;

endTell;

getEmployee #mesquite.charMatrices.BasicDataWindowCoord.BasicDataWindowCoord;

tell It;

showDataWindow #6087454669693722133 #mesquite.charMatrices.BasicDataWindowMaker.BasicDataWindowMaker;

tell It;

getWindow;

tell It;

setExplanationSize 30;

setAnnotationSize 20;

setFontIncAnnot 0;

setFontIncExp 0;

setSize 1383 751;

setLocation 118 47;

setFont SanSerif;

setFontSize 10;

getToolPalette;

tell It;

setTool mesquite.charMatrices.ColorCells.ColorCells.ColorCells;

endTell;

setTool mesquite.charMatrices.ColorCells.ColorCells.ColorCells;

colorCells #mesquite.charMatrices.NoColor.NoColor;

colorRowNames #mesquite.charMatrices.TaxonGroupColor.TaxonGroupColor;

colorColumnNames #mesquite.charMatrices.CharGroupColor.CharGroupColor;

colorText #mesquite.charMatrices.NoColor.NoColor;

setBackground White;

toggleShowNames on;

toggleShowTaxonNames on;

toggleTight off;

toggleThinRows off;

toggleShowChanges on;

toggleSeparateLines off;

toggleShowStates on;

toggleReduceCellBorders off;

toggleAutoWCharNames on;

toggleAutoTaxonNames off;

toggleShowDefaultCharNames off;

toggleConstrainCW on;

toggleBirdsEye off;

toggleColorOnlyTaxonNames off;

toggleShowPaleGrid off;

toggleShowPaleCellColors off;

toggleShowPaleExcluded off;

togglePaleInapplicable on;

togglePaleMissing off;

toggleShowBoldCellText off;

toggleAllowAutosize on;

toggleColorsPanel off;

toggleDiagonal on;

setDiagonalHeight 80;

toggleLinkedScrolling on;

toggleScrollLinkedTables off;

endTell;

showWindow;

getWindow;

tell It;

forceAutosize;

endTell;

getEmployee #mesquite.charMatrices.AlterData.AlterData;

tell It;

toggleBySubmenus off;

endTell;

getEmployee #mesquite.charMatrices.ColorByState.ColorByState;

tell It;

setStateLimit 9;

toggleUniformMaximum on;

endTell;

getEmployee #mesquite.charMatrices.ColorCells.ColorCells;

tell It;

setColor Red;

removeColor off;

endTell;

getEmployee #mesquite.categ.StateNamesEditor.StateNamesEditor;

tell It;

makeWindow;

tell It;

setExplanationSize 30;

setAnnotationSize 20;

setFontIncAnnot 0;

setFontIncExp 0;

setSize 1383 751;

setLocation 118 47;

setFont SanSerif;

setFontSize 10;

getToolPalette;

tell It;

setTool mesquite.categ.StateNamesEditor.StateNamesWindow.ibeam;

endTell;

rowsAreCharacters on;

toggleConstrainChar on;

toggleConstrainCharNum 3;

togglePanel off;

toggleSummaryPanel off;

endTell;

showWindow;

endTell;

getEmployee #mesquite.categ.StateNamesStrip.StateNamesStrip;

tell It;

showStrip off;

endTell;

getEmployee #mesquite.charMatrices.AnnotPanel.AnnotPanel;

tell It;

togglePanel off;

endTell;

getEmployee #mesquite.charMatrices.CharReferenceStrip.CharReferenceStrip;

tell It;

showStrip off;

endTell;

getEmployee #mesquite.charMatrices.QuickKeySelector.QuickKeySelector;

tell It;

autotabOff;

endTell;

getEmployee #mesquite.charMatrices.SelSummaryStrip.SelSummaryStrip;

tell It;

showStrip off;

endTell;

getEmployee #mesquite.categ.SmallStateNamesEditor.SmallStateNamesEditor;

tell It;

panelOpen true;

endTell;

endTell;

endTell;

endTell;

end;
